# On the road to fully automated insulin delivery: A systematic review of meal announcement free algorithms

**DOI:** 10.1371/journal.pdig.0001492

**Published:** 2026-07-09

**Authors:** Muhammad Ibrahim, Aleix Beneyto, Ivan Contreras, Josep Vehi

**Affiliations:** 1 Modeling and Intelligent Control Engineering Laboratory (MICELab), Institut d’Informàtica i Aplicacions, Universitat de Girona, Girona, Spain; 2 Centro de Investigación Biomédica en Red de Diabetes y Enfermedades Metabólicas Asociadas (CIBERDEM), Instituto de Salud Carlos III, Madrid, Spain; Xinjiang Medical University Affiliated First Hospital, CHINA

## Abstract

Fully automated insulin delivery (FAID) systems aim to regulate blood glucose levels in individuals with type 1 diabetes with minimal user input. A key challenge to achieving full automation is the need for manual meal announcements. This review focuses on algorithms developed to detect and compensate for unannounced meals. A systematic literature search was conducted across PubMed, Scopus, IEEE Xplore, and Web of Science (2000 – 2025), and the review was reported in accordance with PRISMA guidelines. From 1205 initially retrieved articles, 69 studies met predefined eligibility criteria and were included in the final review. Extracted data included algorithm type, data source (in-silico or clinical), performance metrics, and glycemic outcomes where available. The reviewed algorithms include heuristic rule-based methods, machine learning models, and control systems theory approaches. While varying in complexity and detection strategies, these approaches aim to identify meal events and estimate carbohydrate intake for timely insulin dosing. Median performance across studies included a sensitivity of 88%, precision of 93%, and detection times ranging from 25 to 40 minutes. In-silico evaluations generally reported more false positives than in-vivo. Reported glycemic outcomes demonstrated time-in-range values between 65% and 89%, highlighting the potential of these systems to support fully autonomous glucose regulation. Automated meal detection and compensation algorithms show promise for integration into FAID systems, with encouraging detection performance and glycemic outcomes. However, the evidence remains heterogeneous, and a substantial portion is still based on in-silico studies rather than clinical validation. Challenges remain in minimizing false positives, ensuring generalizability across populations, and validating performance in real-word settings.

## Author summary

Living with type 1 diabetes means constantly balancing insulin and food intake. Even with the help of modern automated insulin delivery systems, people still need to announce their meals by entering carbohydrate information into the device. This step is often skipped or estimated incorrectly, which can cause dangerous swings in blood sugar.

In our review, we looked at research published between 2000 and 2025 on algorithms that aim to detect meals automatically and adjust insulin without user input. We examined 69 studies, ranging from simple rule-based methods to advanced machine learning and control strategies. These approaches use signals like continuous glucose monitoring, insulin delivery records, and sometimes wearable sensors to recognize eating events.

We found that many algorithms can detect meals reliably and help keep glucose levels in a safer range, though real-world performance still varies. By bringing together results from both simulations and clinical trials, our review shows what progress has been made and what challenges remain. We hope this work will support the development of safe, user-free systems that reduce the daily burden of diabetes management.

## 1. Introduction

Type 1 diabetes (T1D) is characterized by the absence of endogenous insulin production, requiring lifelong insulin therapy to prevent hypoglycemia and hyperglycemia [1]. Multiple daily injections and continuous subcutaneous insulin infusion have improved outcomes, but both demand frequent adjustments and impose a considerable cognitive burden. Automated insulin delivery systems, developed since the 1960s to integrate continuous glucose monitoring (CGM), insulin pumps, and control algorithms, aim to reduce this burden by automating insulin dosing [2]. A key obstacle to full automation, however, is the need for manual meal announcements. Detecting unannounced meals remains one of the most complex challenges in advancing fully closed-loop automated insulin delivery (FAID) systems.

An early staged roadmap for automated insulin delivery outlined the progression from safety features such as low-glucose insulin suspension, to predictive algorithms for managing glycemic excursions, and later to hybrid closed-loop (CL) systems requiring user input for meals and activity [3]. These hybrid CL systems form the basis of most current commercial products, automating basal insulin delivery but still relying on user-announced meals. This dependence introduces errors, as individuals often misestimate or omit carbohydrate (CHO) entries, leading to impaired postprandial glucose control. The final stages of the roadmap envisioned systems with minimal user intervention, incorporating advanced control strategies and, in some cases, multi-hormonal designs. Within this context, eliminating manual meal announcements has become a central focus in the development of FAID technologies.

**Fig 1:**
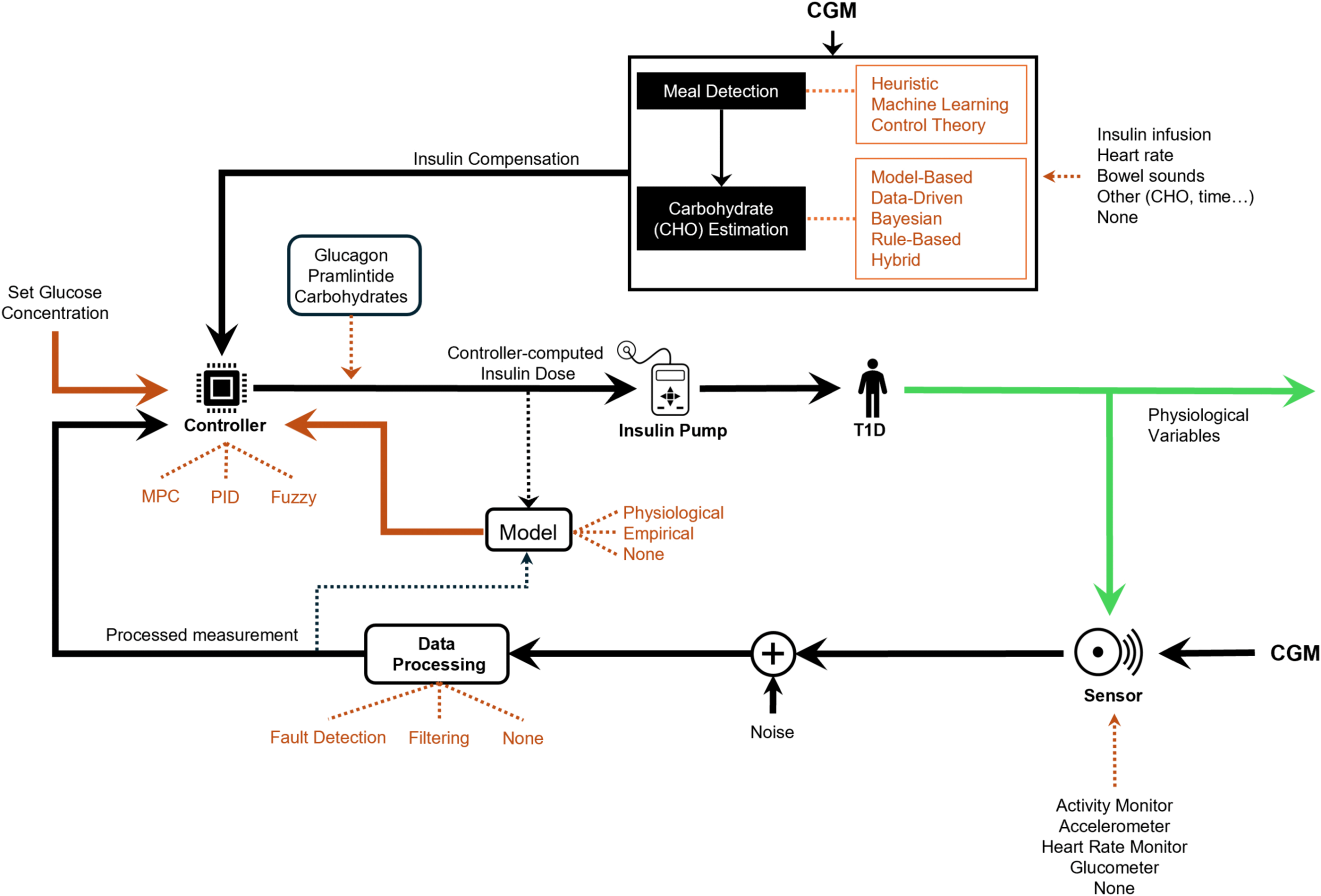
Taxonomy of a fully automated insulin delivery system with automated meal detection, adapted from [3]. Black lines denote operational information and action flows, green lines represent physiological variables, and orange lines indicate configurable design elements. Solid lines show core connections present across all fully automated insulin delivery system configurations, whereas dotted lines indicate optional connections that may vary depending on system design, algorithm choice, or automation level.

Fig 1 presents an updated taxonomy of FAID systems. The schematic distinguishes physiological variables, such as blood glucose and heart rate, from digitally measured or processed signals, while also separating the main operational information and action flows from configurable design elements. These configurable components may differ across implementations, for example in controller type, sensing inputs, or estimation strategy.

**Fig 2:**
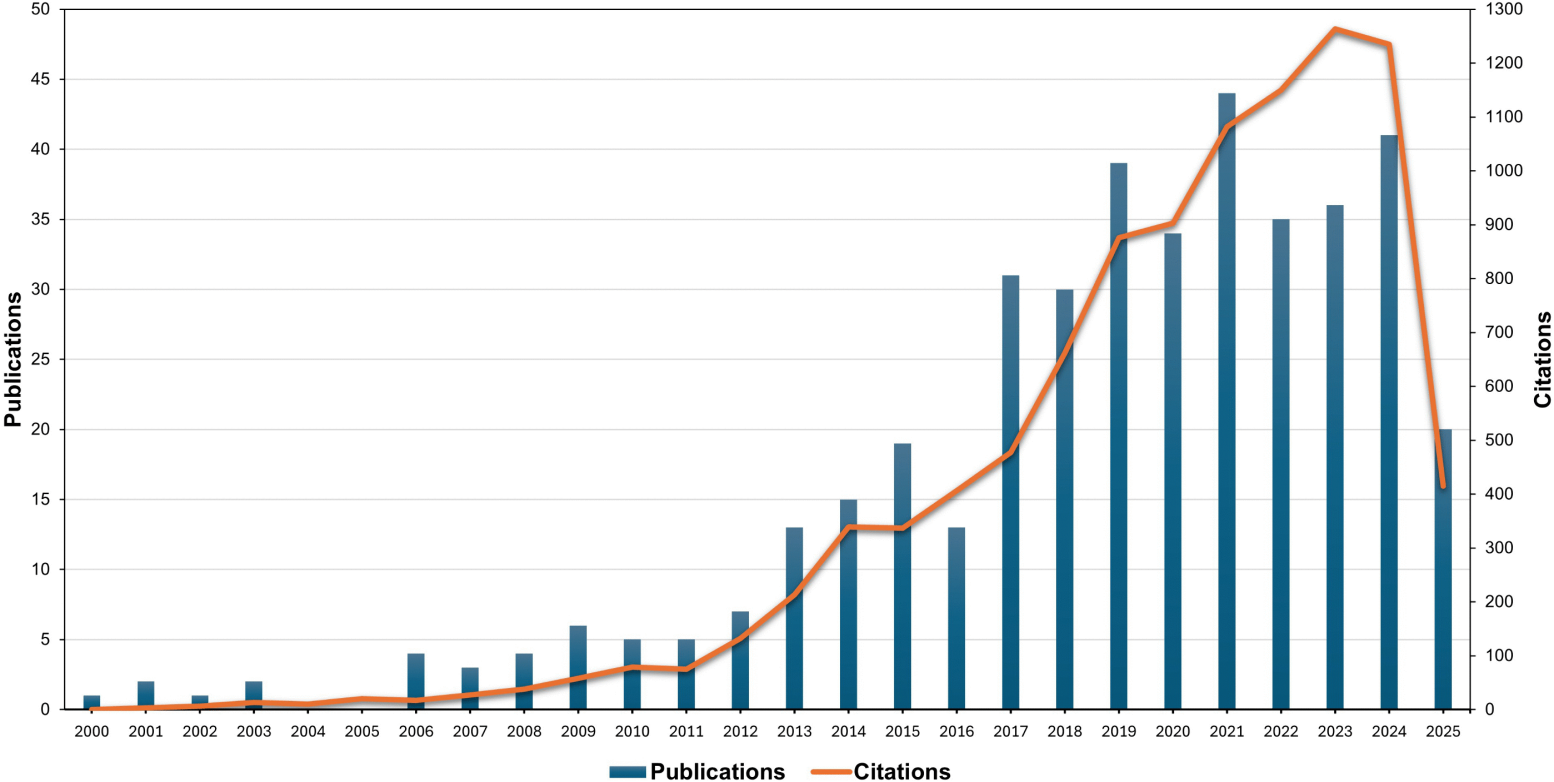
Published articles related to fully automated insulin delivery (report generated via Web of Science in July 2025).

Achieving FAID without manual intervention remains challenging because of the variability in meal timing, size, and composition. These difficulties have driven increasing research attention, as illustrated in Fig 2, which shows the steady rise in publications addressing this problem. Research on unannounced meals has produced two main types of algorithmic strategies: implicit and explicit. Implicit approaches infer meals indirectly from observed glucose and insulin patterns without explicitly identifying the event [4–6], whereas explicit methods aim to directly detect meals and estimate CHO content to guide insulin delivery [7–9]. Most algorithms have been validated initially in simulation studies, with several advancing to clinical evaluation [10,11]. Recent findings highlight encouraging progress, suggesting that some of these methods are moving closer to practical implementation in FAID systems.

The clinical relevance of meal detection algorithms depends not only on whether meals can be detected, but also on how detection performance affects insulin delivery and glycemic control. For example, one in-silico study [12] showed that high detection performance, combining high sensitivity and precision with low false-positive (FP) burden, was associated with improved glycemic outcomes compared with hybrid CL, with higher time in range (TIR) and lower time above range (TAR). In contrast, another study [13] reported a meal detection time (DT) of 40 min, yet FAID resulted in lower TIR and higher TAR than announced-meal hybrid CL, suggesting that delayed detection can limit postprandial benefit despite successful meal recognition. FPs are also clinically important because they contribute to the broader error profile of the meal detection algorithm and may compromise glycemic outcomes when combined with delayed or inaccurate detection. For example, one in-silico study [14] reported non-zero FPs and a DT of 45 min, with lower TIR and higher time below range (TBR) and TAR than basal-bolus control with meal announcements. For this reason, understanding the different algorithmic strategies requires considering both their technical design and their potential implications for safety and clinical outcomes.

This review examines algorithmic strategies for managing unannounced meals in FAID. It considers both foundational work and recent advances, with emphasis on methods that reduce or eliminate the need for manual meal announcements. The remainder of the paper is organized as follows: Section 2 outlines the methodology for identifying relevant studies. Section 3 summarizes algorithmic approaches, performance metrics, and data sources. Section 4 provides a critical discussion of the findings, limitations, and open challenges. Section 5 concludes with perspectives on future directions.

## 2. Methodology

A systematic literature search was conducted in August 2025 to identify relevant publications on automated meal detection and compensation algorithms within the context of FAID systems for individuals with T1D. Four scientific databases were queried: PubMed, Scopus, IEEE Xplore, and Web of Science.

The search strategy employed a comprehensive set of keywords covering three main thematic areas: (i) meal-related terminology (meal, intake, uptake, eating), (ii) detection and estimation processes (detect, monitor, measure, assess, estimate), and (iii) algorithmic and computational approaches (artificial pancreas, automated insulin delivery, algorithm, machine learning, artificial intelligence, neural networks, deep learning, Kalman, observer, control, decision rules, fuzzy). Boolean operators were used to construct combinations of these terms for maximum sensitivity. The full search string used in each database is provided in Table 1.

**Table 1:** Search query used and total number of collected papers.

Search Query	Database	Result
(meal OR intake OR eating OR uptake) AND (detect OR monitor OR measure OR assess OR estimate) AND (“artificial pancreas” OR “automated insulin delivery”) AND (algorithm OR “artificial neural network” OR “deep learning” OR “machine learning” OR “artificial intelligence” OR ai OR observer OR kalman OR control OR “decision rules” OR fuzzy)	PubMed	345
	Scopus	237
	IEEE Xplore	205
	Web of Science	418
**Total Articles**		**1205**

Only articles written in English and published from January 2000 to August 2025 were considered. Eligible studies had to describe algorithmic methods specifically designed to detect or compensate for unannounced meals in individuals with T1D, and report on performance using quantitative metrics. The review included both journal and conference papers that underwent peer review. Non-peer-reviewed conference records or listings without full methodological and quantitative reporting were excluded. Studies were excluded if they did not present methodological details, lacked any performance evaluation, or were not focused on postprandial glycemic control.

**Fig 3:**
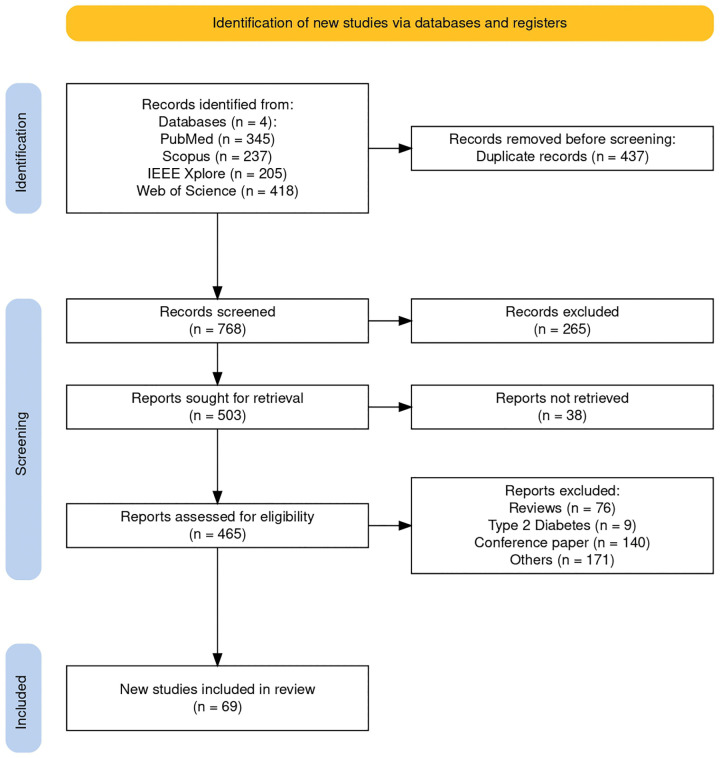
Flowchart based on PRISMA guidelines outlining inclusion and exclusion criteria.

This review was conducted using a systematic methodology aligned with PRISMA guidelines [15], and the completed PRISMA 2020 checklist is provided in S1 Checklist. A formal review protocol was not preregistered for this study, which we acknowledge as a limitation regarding methodological transparency. The review was designed to synthesize and compare algorithmic developments in meal detection and compensation across heterogeneous study designs, rather than to estimate a pooled intervention effect. Initial screening was carried out by evaluating the titles and abstracts, and studies that clearly did not meet the inclusion criteria were excluded. Study screening and eligibility assessment were conducted by one reviewer (first author), with decisions reviewed in consultation with the supervising authors where necessary to ensure consistency. Additionally, the reference lists of all included studies were manually screened to identify any relevant publications not captured by the database search. The initial query across four databases yielded 1205 records. After the removal of 437 duplicates, 768 unique articles remained for screening. Following title and abstract screening, 503 articles were selected for full-text retrieval, of which 38 could not be accessed. A total of 465 articles underwent full-text assessment for eligibility. Ultimately, 69 studies met all inclusion criteria and were incorporated into the final review. The excluded studies included 76 reviews, 9 focused on type 2 diabetes, and 311 others, which comprised non-peer-reviewed conference records, preprints, posters, clinical trials, and other articles that did not meet the inclusion criteria, including insufficient methodological detail, lack of quantitative performance evaluation, or lack of clearly presented results. The complete article selection process is summarized in Fig 3.

From each selected study, the following information was extracted: author and year, methodological description of the proposed approach, data source and type (in silico, clinical, or real-world), cohort size and characteristics (if applicable), type of therapy (single-hormone or dual-hormone), and whether the algorithm included additional modules (e.g., meal size estimation or activity compensation). Reported performance metrics were also recorded. Data extraction was performed by the first author, with extracted data checked by the supervising authors. Interpretation of findings was conducted collaboratively. Given the heterogeneity across included studies in datasets, validation environments, system configurations, and reported performance metrics, the results were compared and interpreted qualitatively rather than statistically pooled in a formal meta-analysis. A formal risk-of-bias or certainty-of-evidence assessment was not performed, as the included studies primarily focused on algorithm development, simulation environments, and engineering performance metrics. Standard risk-of-bias tools for clinical or interventional studies were therefore not applicable to this body of evidence.

For comparative analysis, the following performance indicators were considered: true positives (TP), FP, false negatives (FN), sensitivity, specificity, accuracy, precision, F1-score, false positives per day (FP/day), and DT. When possible, normalized metrics (FP/day) were derived from reported results and used as the primary basis for cross-study comparison of FP burden. FP/patient/day was not used uniformly because some studies, especially in-silico studies, reported only total evaluation days and not clearly defined patient-level monitoring time, making this conversion unreliable in some cases. In studies that incorporated meal size estimation, CHO estimation error was also extracted. For algorithms that included an insulin compensation component, postprandial glycemic outcomes such as TBR, TIR, and TAR were documented where available.

## 3. Results

Various methodologies have been employed in pursuit of FAID system. Some studies have utilized explicit modules for automated meal detection, estimating meal content size, and adjust the therapy accordingly. Other studies have addressed meal disturbances implicitly by observing changes in blood glucose (BG) levels. The algorithms reviewed have been broadly classified based on their methodologies into heuristic approaches, machine learning approaches, and control systems theory approaches. Heuristic approaches typically involve rule-based systems that use predefined thresholds to detect meal events. Machine learning approaches leverage data-driven models to identify patterns and predict meal occurrences. Control systems theory approaches use mathematical models and observers to manage disturbances such as unannounced meals. In this section, we will discuss the methodologies used in the shortlisted papers.

### Heuristic approaches

Heuristic approaches to meal detection and size estimation rely on predefined thresholds or decision rules derived from empirical knowledge or expert insights. Their main strengths are simplicity and ease of implementation: they require relatively low computational resources and can be deployed quickly. At the same time, they are limited in flexibility, often struggling to account for individual variability or unexpected eating behaviors. As a result, these methods are best suited to settings where dietary patterns are relatively uniform and predictable.

To improve performance, feedback mechanisms are sometimes incorporated to adjust the rules based on observed outcomes. This iterative refinement allows the system to adapt more effectively to user-specific profiles or contextual factors, enhancing both detection and estimation accuracy over time.

#### Decision rules.

Heuristic approaches to meal detection typically follow a two-step process: first, relevant signals are selected and preprocessed; second, informative features are extracted and used within a rule-based framework. These approaches generally rely on comparisons or thresholds applied to features such as the BG rate of change (RoC) or the area under the curve.

A voting-based system was introduced in [7], combining RoC features derived from both raw CGM data and Kalman filter (KF) estimates. The method evaluates backward differences of raw glucose, KF-estimated glucose, and its RoC, each compared against patient-specific thresholds (RoC, maximum RoC, glucose level, acceleration). A meal is detected if two of three or three of four criteria are exceeded within a 5-min window. Similarly, [16] triggers detection when the RoC crosses a predefined threshold, prompting insulin delivery, while [17] applies a bang-bang controller that toggles insulin on or off based on glucose derivative thresholds.

Other studies [18,19] exploit first- and second-order derivatives of BG. A meal is detected when the conditions in Equation 1 are satisfied (sampling interval 5 min), where $y_{k}$ denotes the glucose measurement at sampling instant k; meal size is then estimated using a finite impulse response filter:


$$\begin{array}{c} \left\{ \begin{array}{c} \Delta y_{k}\geq 1.2\;and\;\Delta^{2}y_{k-1}<0.45\;and\;\Delta^{2}y_{k}\geq 0.45,\\ \Delta y_{k}\geq 1.5\;and\;\Delta^{2}y_{k-1}<0.45. \end{array}\right. \end{array}$$ (1)

In [20] and [13], detection is based on the cumulative sum test together with a CHO rate threshold. In [21], two threshold-based methods were proposed: one applying a threshold to the current glucose rate of appearance estimate, and another employing the glucose rate increase detector algorithm. The same approach is also reported in [22,23], and [24]. This algorithm proceeds in three stages: preprocessing to filter CGM noise, estimation of glucose RoC, and detection when the RoC consistently exceeds a predefined threshold. When such a condition is met, a meal is inferred and an insulin bolus is delivered to mitigate hyperglycemia.

Other variations of rule-based detection include the method in [25], which detects meals by calculating the Euclidean distance between predicted and measured BG while also considering insulin delivery and physical activity data (e.g., heart rate). In [26], a minimal glucose–insulin model is used to compare CGM sub-windows through energy ratios. Sequential rules then generate a decision score, and a meal is detected once the score exceeds a predefined threshold. This method is physiologically invariant and operates consistently across patients without the need for personalization.

#### Fuzzy logic.

Fuzzy logic provides a more flexible alternative to simple threshold-based rules by allowing inputs to be represented as fuzzy sets rather than fixed values. Each input is assigned a degree of membership to categories through membership functions, often defined using glucose levels and their derivatives. These sets are then combined through mathematical operations and translated into inference rules. The resulting weighted outputs determine whether a meal event is detected.

In [27], two control strategies were combined: a fuzzy logic controller (control-to-range) and a control-to-target approach, where the latter refined insulin dosing recommendations based on the former. In [28], a fuzzy logic–based system was developed to automatically identify unannounced meals and adjust insulin delivery, evaluated with both standard and faster insulin aspart CL systems. Study [29] incorporated BG levels and their first and second-order derivatives, categorized into fuzzy sets via numerical differentiation and RoC-based membership functions. Study [30] also applied fuzzy inference principles to meal detection. Finally, [31] presented a pilot study assessing the safety and efficacy of fuzzy logic control in a research setting, considering hormonal variations and unannounced small to medium-sized meals.

Some algorithms combine features of both fuzzy logic and decision rules, making them difficult to assign to a single category. For example, [30] and [32] map glucose RoC derivatives into fuzzy sets, but the final detection step relies on weighted inference rules. In these cases, fuzzy logic is applied to interpret signal dynamics, while the decision-making process follows a rule-based structure. Such approaches can be considered hybrid, as they integrate fuzzy preprocessing with rule-based classification.

### Machine learning approaches

Machine learning methods are increasingly applied in the development of FAID systems. Their applications include glucose prediction, optimization of insulin delivery, and event detection such as meals and exercise [33]. A range of techniques have been explored, including time series models, binary classifiers, and reinforcement learning. These methods provide flexibility in capturing nonlinear and individualized patterns.

#### Time series detection algorithms.

Time series data such as CGM readings, insulin delivery records, and activity logs are central to FAID systems because of their sequential nature and temporal dependencies. Recurrent neural networks, particularly long short-term memory (LSTM) architectures, are widely used as they can capture glucose dynamics beyond the capability of threshold-based methods.

In [34], LSTM-based networks combined with ensemble strategies classified sequences of CGM measurements to detect meal disturbances. In [35], personalized LSTM models predicted upcoming mealtimes from insulin pump data, processing 48-hour sequences in 30-minute intervals and using peak detection on model outputs. This approach outperformed standard baselines and autoregressive models, even among individuals with irregular eating patterns.

Other studies have combined CGM, insulin, and meal information. A sequence-to-sequence LSTM architecture for multitask quantile regression predicted short-term glucose trajectories and flagged unannounced meals when observations fell outside the 95% prediction interval [9]. The system then applied an iterative search to estimate meal size, guided by mean absolute error (MAE). Similarly, [36] employed two deep neural networks, one for binary meal detection using a Sigmoid output layer and another for meal size estimation with a ReLU output.

Expanding beyond CGM, [37] applied multiple recurrent neural network variants (standard LSTM, 1D LSTM, 2D ConvLSTM, and Bi-LSTM) for joint detection of meals and exercise, classifying events into four categories: neither, exercise only, meal only, or both. In [38], wrist motion data from accelerometers and gyroscopes were used with personalized LSTM models to detect food intake. Data were segmented into one-minute windows and labeled by self-reported meals, with models trained per participant using an 80/20 temporal split.

#### Binary classification algorithms.

Meal detection can also be framed as a binary classification problem, where the task is to determine whether a meal has occurred. Tree-based models, including random forests and boosted trees, are widely used in this context. These models combine the outputs of multiple decision trees to improve accuracy. They typically rely on physiological and behavioral features such as glucose levels, heart rate, physical activity, skin temperature, and respiration rate, which often change after food intake.

Other classification techniques have also been applied. An isolation trees algorithm was used in [39] for unannounced meal detection, while linear discriminant analysis [21], multi-layer perceptron, Naïve Bayes, and logistic regression have been tested in several studies [40,41]. Ensemble methods have shown particular promise. For example, [11] combined multi-layer perceptron, random forests, and logistic regression to improve detection across meals of varying absorption rates and sizes. Neural networks have also been used for meal detection [10], using a dual-branch architecture with one branch dedicated to binary meal detection and the other to meal size estimation through multiclass classification. Similarly, [36] used deep neural networks to classify meal intake.

Comparative evaluations have further expanded the range of models tested. In [42], decision trees, random forests, support vector machines, Gaussian and complement Naïve Bayes, feedforward neural networks, LSTM networks, and threshold-based methods were benchmarked across in-silico cohorts representing adults, adolescents, and children. Beyond conventional data sources, alternative modalities have also been explored. Abdominal sound recordings were analyzed in [43], where features extracted from bowel sounds were classified with a support vector machine, highlighting the potential of audio-based monitoring as a non-invasive approach to meal detection. In a related study [44], individualized postprandial glucose response patterns were modeled in free-living conditions, with candidate meal events classified using AdaBoost, random forests, and decision trees trained per participant.

#### Reinforcement learning.

Reinforcement learning is a branch of machine learning that trains agents to make sequential decisions by interacting with an environment. The agent learns to map observed states to actions that maximize a cumulative reward signal over time. In FAID systems, reinforcement learning is particularly useful for optimizing insulin dosing under uncertainty and patient variability, as it adapts dynamically to the outcomes of prior actions.

In [45], an implicit method called Bioinspired was developed for automated insulin infusion using reinforcement learning. The approach incorporates reward functions to capture the temporal homeostatic objective and discount factors to reflect individual pharmacological characteristics. It was evaluated on virtual patients in a simulation environment with unannounced meal intakes. More recently, [46] proposed a FAID system that leverages deep reinforcement learning to compute personalized insulin boluses without requiring CHO estimation or manual meal announcements. Building on earlier unscented KF-based meal detection strategies [8], this system integrates a deep reinforcement learning agent trained to optimize glycemic outcomes by learning insulin dosing policies in response to detected unannounced meals.

### Control systems theory

Control systems theory provides the foundation for FAID development by enabling real-time regulation of BG through continuous monitoring and adaptive insulin delivery. These controllers can adapt to physiological variability. Strategies range from linear designs to advanced nonlinear approaches, reflecting the flexibility of control theory in managing complex biological systems.

#### Disturbance observer-based control.

In addition to the primary process variable, control systems must account for disturbances that can compromise performance. In diabetes, meal intake is a major disturbance, often unpredictable and unannounced. Disturbance observer–based control methods are designed to estimate and mitigate such effects [47].

Several observer-based approaches have been proposed for meal management. In [48], a disturbance observer–based control system estimates glucose disturbances from CGM data, insulin infusion rates, and subject-specific models. A related study [49] integrated a moving horizon state estimator into an automated insulin delivery system, showing faster insulin adjustments and improved postprandial control compared with a Luenberger observer, without increasing hypoglycemia risk. In [50], an extended state observer was applied within a dual-hormone (insulin and glucagon) system. Here, meal detection is implicit: deviations from predicted glucose trajectories are interpreted as disturbances, prompting corrective insulin or glucagon dosing.

Sliding mode control represents another robust disturbance observer–based control technique, valued for suppressing parameter variability and external disturbances [47]. In [51], a first-order sliding mode observer was used to estimate the rate of glucose appearance, and a super-twisting observer was developed to detect meals, framing the task as a fault detection problem. A recent extension [52] combined this approach with nonlinear switching logic, enabling coordinated actions for insulin delivery, rescue CHO administration, and insulin-on-board limitation. The method from [51] was further advanced in [53] by integrating a super-twisting residual generator with a Kalman filter to estimate BG derivatives, paired with decision rules for meal detection, and validated using clinical data.

#### Model predictive control.

Model predictive control (MPC) has been widely explored for FAID, as it anticipates glucose trajectories and adjusts insulin delivery proactively, helping to mitigate delays in insulin absorption. Several extensions have been developed to improve performance during unannounced meals. In [54], MPC was combined with an automatic bolus priming system that detects disturbances by fitting a polynomial to recent CGM data and applying logistic regression. When the probability of a disturbance is high, a priming bolus is delivered and scaled according to both the disturbance likelihood and the patient’s total daily insulin. This approach, further extended in multistage MPC, anticipates eating behaviors from historical data. Similar strategies were applied in [55,56] (known as RocketAP system), and [57]. A probabilistic extension, multiple model probabilistic predictive control, was later proposed in [58–60]. In addition to forecasting mean glucose trajectories, this approach also predicts uncertainty in future BG levels, enabling more robust responses to unannounced meals and improving safety.

Zone-MPC is another variant, designed to maintain glucose within a predefined range while reducing pump activity and power consumption. In [61], zone-MPC was integrated with a health monitoring system to enhance safety during unannounced meals, overnight periods, and physical activity. The health monitoring system provided an additional safeguard by monitoring risks such as hypo- or hyperglycemia and generating alerts to users or caregivers.

Finally, [62] introduced an extended model predictive control strategy that combines predictive modeling with a risk management framework. By incorporating prediction uncertainty and the asymmetric risks of hypo- and hyperglycemia, the proposed controller dynamically adjusts insulin delivery to optimize safety. This approach anticipates the impact of unannounced meals and controller actions, improving both robustness and efficacy in glucose management.

#### State estimation and control.

Accurate estimation of hidden physiological states is critical for effective glucose regulation in automated insulin delivery systems, particularly when meals are unannounced. Many approaches model meals as unmeasured disturbances and use observers to infer their effects on glucose dynamics. Commonly applied observers include the Luenberger observer, Kalman filter (KF), unscented KF, and extended KF [7,18–20,53,63].

State estimators are often combined with statistical tests or decision rules to improve meal detection. For example, [20] and [13] combined a KF with a cumulative sum test applied to the residuals between expected and actual glucose values to detect and estimate meal intake. The unscented KF has also been used for state estimation in several studies, including the meal detection methods in [64] and [65]. In [64], a modified Bergman minimal model was used to enable simultaneous estimation of states and parameters. In [65], unscented KF served as a detection module within a multivariate adaptive FAID system, where detection automatically triggered meal bolus delivery. In [8] and [66], a hybrid approach combined unscented KF with sliding window and cross-covariance analysis, detecting meals when the covariance exceeded a threshold, BG slope was positive, and detection occurred during daytime. Similarly, [12] paired unscented KF with decision rules based on CGM thresholds, forward glucose differences, and residual errors.

Other strategies extend estimation into the frequency or transfer-function domain. In [67], transfer functions were used to describe glucose responses to insulin and meal disturbances, with a controller acting on deviations from a reference signal. Building on this, [68] proposed a feedback-based method to estimate meal size, where the difference between predicted and observed BG was treated as an error signal to refine carbohydrate intake estimates in multiple stages. An improved version [69] added a KF to increase accuracy and a saturation block to prevent non-physiological outputs.

Moving horizon estimation has also been explored. In [70], it was used to detect unannounced meals and estimate carbohydrate intake rates. A refined version, termed committed moving horizon estimation, was proposed in [71], combining multiple estimator instances to balance past and future information at the decision point, improving the timeliness and precision of detection. Similarly, [14] introduced a variable state-dimension KF that switched between quiescent and maneuvering modes depending on BG thresholds, allowing more accurate estimation of meal-induced variability.

Sensor redundancy and adaptive filters have been investigated as well. In [72], dual CGM sensors were paired with individual unscented KFs, and anomalies were cross-validated to distinguish meals from sensor faults. In [73], a personalized autoregressive moving-average model with KF was used for real-time BG prediction; deviations from forecasts triggered alarms for unannounced meals. Adaptive estimation was also employed in [74], where KF combined with maximum a posteriori parameter estimation and likelihood ratio tests to detect disturbances. The same method was later applied in a multihormonal system incorporating insulin and pramlintide [75]. Additional works [76,77], and [78] used KFs to compare expected and measured BG trajectories, while [79] compared glucose predictions from models with and without meal inputs. When a meal-based model gave a closer match to sensor data, the system identified a meal and estimated its size. In [80], an adaptive control system integrated CGM, energy expenditure, galvanic skin response, and insulin-on-board to adjust for both meals and physical activity without manual announcements.

Robust control has also been applied to improve resilience against disturbances. In [81], a proportional integral derivative controller with double phase-lead compensation was shown to enhance stability and responsiveness to meal-induced glucose excursions. In [82], a constrained robust regulator with a robust KF leveraged Markovian jump linear systems to dynamically adjust insulin delivery in response to meal disturbances.

The algorithms reviewed represent diverse methodological approaches, each addressing the challenge of unannounced meal detection in different ways. They differ not only in design but also in their effectiveness across varying scenarios. The next section evaluates their performance using key metrics reported in both in-silico and clinical studies.

### Performance of the algorithms

**Fig 4:**
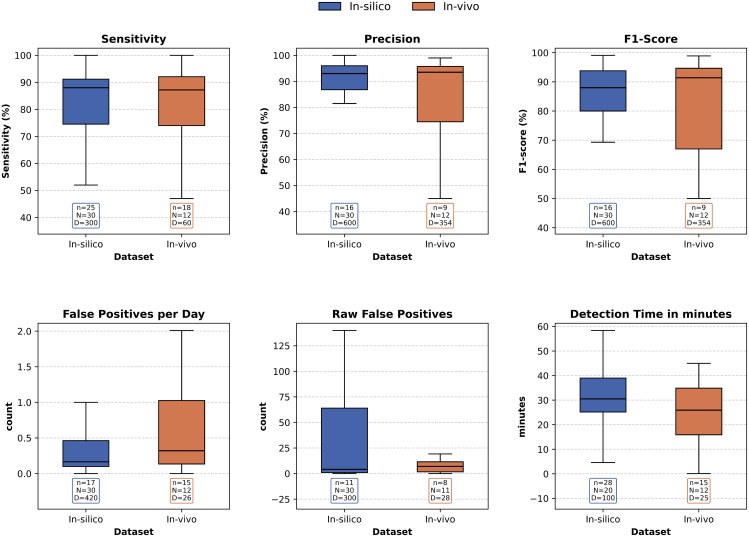
Performance of meal detection algorithms on in-silico vs. in-vivo datasets. Boxplots show study-level distributions for each metric. In each subplot, n denotes the number of unique studies contributing to that metric, while N and D denote the median cohort size and study duration (days), respectively. FP/day is used as the primary normalized metric for cross-study comparison, whereas the raw FP panel is included only for descriptive context based on the values originally reported in the source studies.

**Fig 5:**
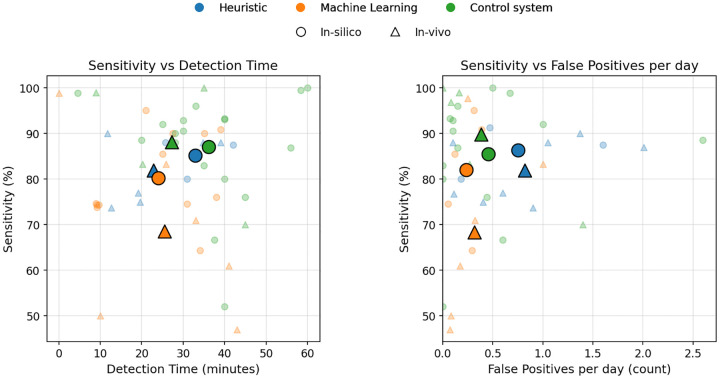
Trade-offs across methods, with large filled markers denoting dataset-specific mean performance.

**Fig 6:**
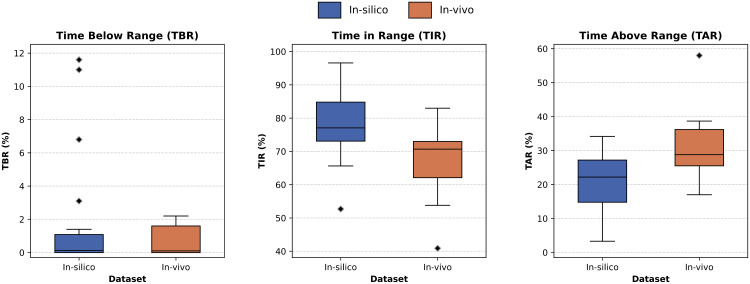
Glycemic outcomes of automated insulin delivery systems with automated meal detection.

**Fig 7:**
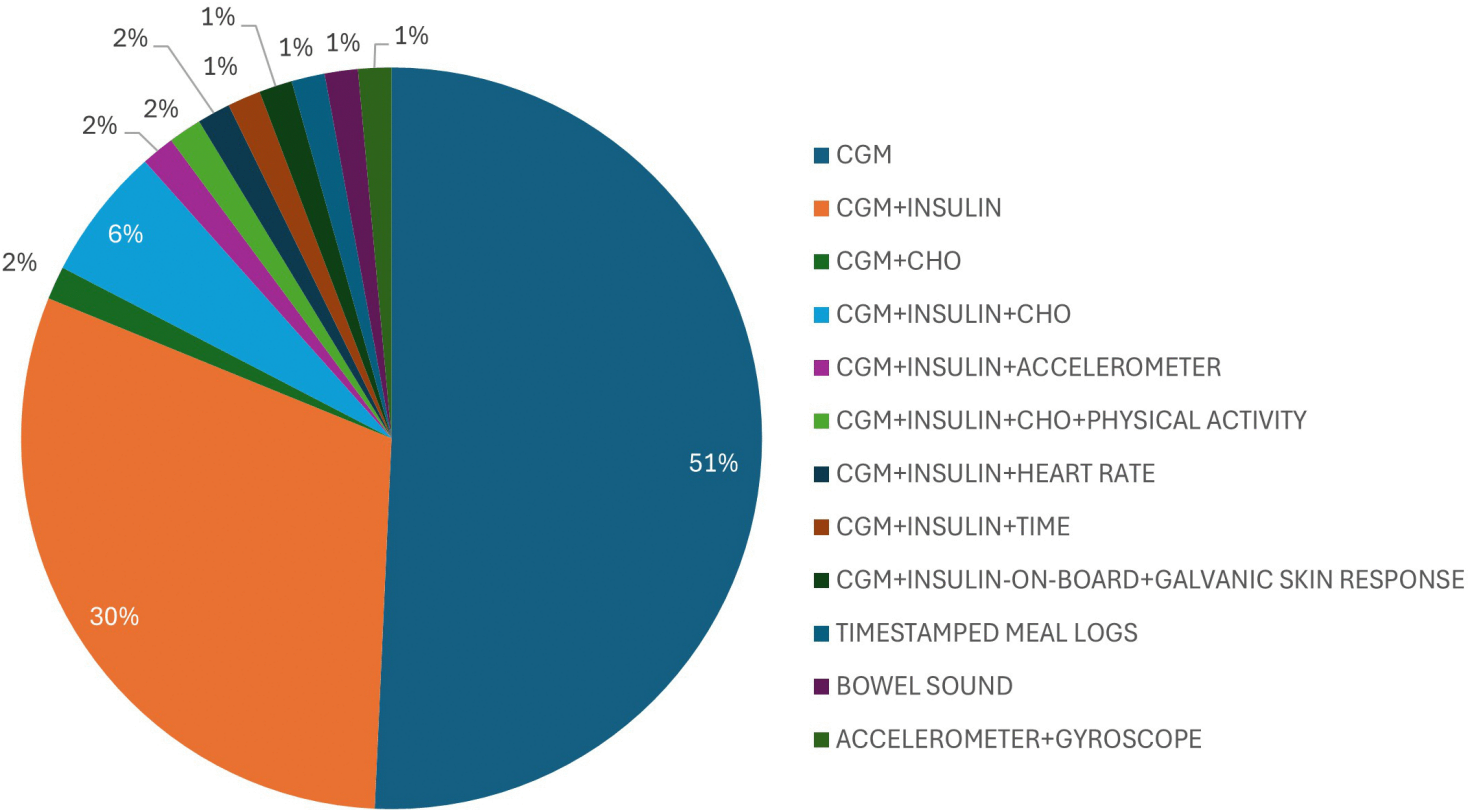
Summary of data used by automated meal detection algorithms.

This section evaluates the performance of all shortlisted algorithms proposed in the literature. The metrics considered include TP, FP, FN, sensitivity, precision, F1-score, FP/day, DT, and TIR. TIR is particularly relevant when meal detection algorithms are integrated with insulin compensation schemes aimed at reducing postprandial excursions. Summary results are shown in Figs 4, 5, 6, and 7, with the following subsections detailing algorithm behavior, detection accuracy, and glycemic outcomes.

#### Performance overview.

The performance of unannounced meal detection algorithms varies considerably depending on methodology, dataset type (in-silico or clinical), and evaluation metrics. Sensitivity is the most frequently reported metric, appearing in 37 of the 69 reviewed studies. It reflects the ability of an algorithm to correctly detect true meal events and serves as a central point of comparison.

Several studies demonstrated strong meal detection sensitivity, particularly those combining control theory with heuristic logic. For example, [12] and [74] reported 100% in in-silico and clinical scenarios, respectively, with very few FP. Likewise, [68] achieved values above 98% across both data types. Ensemble strategies in [11] reached up to 97% in simulation, while deep learning models in [37] maintained values above 90% in real-world validation. These promising results often came with variable FP rates, and some models underperformed in clinical data, underscoring the challenge of generalizability.

Precision provides important context by measuring the proportion of true meal detections among all positive predictions. Studies such as [12] and [37] reported precision values above 95%, indicating high reliability. In contrast, models with high sensitivity but low precision, such as the high-sensitivity configuration in [8], produced excessive FP, reducing their suitability for FAID systems.

The F1-score, which balances sensitivity and precision, offers a useful indicator of overall detection reliability. Several studies reported strong F1-score performance, including [12,38], and [68], with F1-scores exceeding 97% in both in-silico and in-vivo settings, reflecting robust detection performance with limited trade-off between missed events and false alarms. The FP burden remains critical, especially in clinical applications where false detections can lead to unnecessary insulin delivery. However, direct comparison of raw FP counts across studies can be misleading because reported totals depend strongly on cohort size, follow-up duration, and whether results are presented at the study, subject, or simulation level. Therefore, raw FP values should be interpreted descriptively, whereas cross-study comparison is more appropriately based on normalized metrics such as FP/day. For example, low FP/day despite strong overall detection performance was reported in both in-silico and in-vivo settings, including [12] (F1-score 98.6%, 0.5 FP/day), [64] (F1-score 97.6%, 0.08 FP/day), and [51] (F1-score 93.8%, 0.1 FP/day). Likewise, although [68] and [74] reported raw FP totals of 9 and 64, respectively, their FP burden became more interpretable for cross-study comparison when expressed as FP/day (0.16 vs. 0.07), highlighting the importance of denominator-consistent interpretation. These results illustrate the ongoing challenge of balancing sensitivity with specificity across different testing environments.

Mealtime prediction has also been explored as an alternative to detection. The 1-layer LSTM model in [35], trained on insulin pump data from 82 individuals with T1D (43,596 meal logs across 10,497 days), achieved strong results: F1-score of 95.3%, sensitivity of 97.7%, precision of 93.5%, and low normalized error rates (0.25 FP and 0.07 FN per day). Performance was consistent even among participants with irregular routines and stabilized with 60 days of training data. A wearable-based study [38] reported exceptional results using personalized LSTM models with wrist motion data, achieving median F1-score of 99.7%, precision of 99.5%, and sensitivity of 99.4%, demonstrating the feasibility of near real-time detection.

Additional metrics such as specificity and accuracy provide further context. Specificity quantifies correct identification of non-meal periods, reducing the risk of insulin over-delivery. For instance, [68] reported specificity of 97.5% in clinical data, while [11] and [34] reported values above 70%. Accuracy has also been reported, though it can be misleading in imbalanced datasets where non-meal periods dominate. Nevertheless, studies such as [34] and [37] reported accuracy above 90%, suggesting strong overall classification accuracy.

In summary, the most robust algorithms show a balance across metrics: high sensitivity and precision, low FP, and consistent clinical performance. However, inconsistent reporting standards still limit direct comparison across studies.

#### Meal detection times.

Detection time plays a critical role in assessing how quickly an algorithm recognizes a meal event after it occurs, since earlier detection enables more timely insulin adjustment and improved postprandial control. Among the reviewed studies, 36 reported DT values. The average DT was about 29 minutes, though with wide variation. Some systems achieved near real-time performance. For example, [68] reported DTs of 4.6 minutes in silico and 9 minutes in clinical settings, whereas [72] observed delays as long as 58.4 minutes.

Machine learning based methods generally achieved faster DTs. In [34], ensemble strategies reported average DTs around 9 minutes. Similarly, [11] reported DTs of 21–31 minutes in in-silico studies, with slightly longer delays in clinical settings. Deep learning methods in [10,46], and [37] maintained DTs under 30 minutes. A personalized LSTM-based method using wearable sensor data achieved a detection latency of 5–6 seconds, enabling near-instant recognition of eating gestures [38]. While based on motion rather than glucose signals, it highlights the feasibility of ultra-fast detection using time-series learning techniques.

Heuristic and control-based methods typically showed longer delays, ranging from 30 to 45 minutes. For example, [53] reported DT of 45 minutes in clinical testing, while [10] achieved 25.9 minutes in a similar setting. Overall, DTs below 30 minutes are achievable with well-tuned algorithms, particularly in in-silico environments; however, achieving shorter DTs in clinical settings remains a key challenge.

An effective unannounced meal detection algorithm achieves high sensitivity, precision, and F1-score while maintaining low FP and DT. Fig 4 summarizes these results. In vivo studies show greater variability in precision and F1-score, while sensitivity remains consistently high across both environments. In-silico evaluations often report higher FP, although this may partly reflect differences in cohort size, evaluation duration, and study design rather than algorithm performance alone. Accordingly, FP-related differences in Fig 4 should be interpreted descriptively, with FP/day providing a more comparable cross-study reference where available. Average DT was slightly higher in in-silico data (31.1 minutes) than in in vivo (25.5 minutes), but both remained around half an hour. Overall, Fig 4 highlights differences in reported performance across evaluation settings without implying a consistent performance advantage for either environment. Detailed results are provided in S1 Table.

#### Trade-offs across approaches.

Fig 5 illustrates the trade-offs between sensitivity, DT, and FP/day across methodological categories, highlighting that no single approach consistently optimizes all three metrics. Direct cross-study comparison remains inherently limited because the included studies differ substantially in datasets, validation environments, system configurations, and metric definitions. Consequently, the patterns shown in Fig 5 should be interpreted as a structured overview of general trends and trade-offs across methodological categories, rather than as strict head-to-head performance rankings.

In the sensitivity versus DT view, heuristic approaches tend to cluster at moderate detection delays while achieving moderate to high values. This pattern reflects their reliance on explicit thresholds and rule-based logic, which typically require sufficiently clear postprandial glucose dynamics before declaring a meal. As a result, these methods favor stable and predictable behavior but inherently delay detection until glucose excursions become evident. Control system approaches reach similar sensitivity levels but are more frequently associated with longer DT, consistent with disturbance-observer and residual-based designs in which meals are inferred indirectly through sustained deviations between predicted and measured glucose, prioritizing robustness and confirmation over early triggering.

Machine learning approaches span a wider region of the sensitivity versus DT space. Some studies achieve high sensitivity with short DT, while others exhibit longer delays or lower sensitivity, reflecting heterogeneity in model architectures, input features, training strategies, and validation settings. This dispersion indicates that data-driven methods can enable earlier detection by exploiting subtle temporal patterns, but that fast detection is not a guaranteed property of the approach itself.

The sensitivity versus FP/day view reveals a complementary and clinically relevant trade-off. Heuristic and control system approaches generally achieve higher sensitivity, although their dataset-specific mean FP/day values are not consistently lower than those of machine learning approaches. In contrast, machine learning approaches tend to show lower mean FP/day in this comparison, but typically at lower sensitivity. This pattern suggests that improving sensitivity may, in some settings, be accompanied by increased FP/day, underscoring the trade-off between detection performance and false-alarm burden.

Taken together, the two views emphasize that improving sensitivity or reducing DT often shifts the operating point toward increased FP/day. Fig 5 therefore does not identify a single superior methodology, but rather illustrates how different approaches occupy distinct regions of the trade-off space, reflecting different priorities between early detection, robustness, and safety. Within each method category, both in-silico and in-vivo studies are distributed across these regions, indicating that cohort characteristics and evaluation setting can meaningfully influence observed performance, even for similar detection strategies. The within-category variability underlying these trends, including medians and outliers across performance metrics, is further summarized in S1 Fig, which complements the scatter plots by highlighting the spread of reported results across studies.

#### Meal detection with insulin compensation.

A total of 44 studies evaluated glycemic outcomes when meal detection algorithms were integrated with insulin compensation strategies, without requiring user-initiated meal announcements. Reported metrics included TIR (70–180 mg/dL), TBR (<70 mg/dL), and TAR (>180 mg/dL). The primary objective in most cases was not to surpass hybrid systems, but to demonstrate that FAID can achieve clinically acceptable outcomes in the absence of manual inputs. Fourteen studies included real patient data, while the remainder relied on in-silico simulations.

Several clinical trials highlight the potential of these approaches. In a crossover trial with nine participants, [10] compared the proposed FAID system to a hybrid MPC-based system over a 4-hour postprandial period with unannounced meals, reporting a 9.1% increase in TIR and a 10.8% reduction in TAR. In [27], a fuzzy logic-based FAID system was compared with home care using sensor-augmented pump therapy in a 24-hour study involving two participants. The FAID system improved TIR by 2.5% and eliminated TBR, although TAR increased by 12.8%. In [57], 29 participants were monitored across three days of CL use. By Day 3, TIR increased by 9.8% and TAR decreased by 11.0% compared to Day 1 of CL. Relative to open-loop (OL) on Day 1, TIR improved by 13.7% and TAR decreased by 13.1%. In [59], the proposed multiple model probabilistic predictive control algorithm was tested in two small cohorts (n = 4 and n = 6), achieving a 24-hour TIR of 71.1%, with 1.31% TBR and 2.51% TAR-2 (>250 mg/dL). In a subsequent 24-hour inpatient study, [60] compared the FAID approach against OL, reporting a 13.5% increase in TIR and reductions of 2.27% in TBR and 12.25% in TAR. Similarly, in [61], a 24-hour trial with unannounced meals reported a TIR of 71.2%, with 2.2% TBR and 28.8% TAR.

Further inpatient studies provide additional evidence. In [79], 11 adolescents participated in a randomized trial comparing FAID with continuous subcutaneous insulin infusion. The FAID system doubled postprandial TIR (40.9% vs. 20.5%) and reduced TAR (58.0% vs. 79.6%). In [31], 12 participants tested a fuzzy logic-based FAID system in a 24-hour study with two unannounced meals; seven completed the trial. The system achieved 65.0% TIR, 0.1% TBR, and 34.8% TAR. These results were reported relative to predefined clinical glycemic targets, as the study was designed as a feasibility evaluation without a parallel comparator system. In [80], an adaptive control algorithm was evaluated across seven clinical cases involving three subjects over 32–60 hours without meal announcements, achieving an average TIR of 62.1%, TBR of 1.6%, and TAR of 36.2%. The study included an initial OL period for calibration but did not report a formal head-to-head comparator; outcomes are therefore interpreted relative to clinical target ranges and feasibility of sustained fully automated control. In [65], the same algorithm was tested in a 60-hour inpatient CL phase compared to a 24-hour OL phase in seven young adults. The FAID system improved TIR by 7.9% and reduced TBR and TAR by 2.3% and 5.4%, respectively. In [56], the RocketAP system was compared with a hybrid CL system during unannounced meals. RocketAP achieved 30% higher TIR and 30% lower TAR over six hours post-meal, with no increase in TBR. In [74], clinical validation of a model-based FAID reduced postprandial glucose area under the curve by 39% compared to conventional insulin therapy.

Multihormonal systems have also been tested. In [75], nine adults with T1D each completed two 27-hour interventions comparing insulin-only and multihormonal (insulin, pramlintide, glucagon) FAID. The insulin-only system achieved 83.3% TIR and 12% TAR, while the multihormonal system reduced TBR to 0.9% but showed slightly lower TIR (81%) and higher TAR (18%). In [28], a 27-hour study with 20 young adults compared standard versus faster insulin aspart in FAID. TIR was 58.6% with standard insulin and 53.8% with faster insulin, although the latter reduced TAR-2 by 1.9%.

The remaining studies used in-silico cohorts and various simulation environments to assess automated meal detection with insulin compensation. Compared to systems without detection, FAID systems consistently improved TIR and reduced TAR, often without increasing TBR [20,22,51,52,58,69,77], and [13]. Reported gains in TIR ranged from 1.7% to 30%, while TAR reductions ranged from 1.8% to 31%. An exception was noted in [55], where TIR decreased slightly by 1.3% despite a 2.1% reduction in TAR compared to Control-IQ under unannounced meal conditions.

In-silico evaluations of reinforcement learning–based systems also demonstrated effective glycemic regulation. A bioinspired reinforcement learning controller achieved a TIR of 89.6% with a mean glucose of 124.7 mg/dL in response to unannounced meals, showing robustness across variations in insulin sensitivity and dawn phenomenon [45]. Similarly, a deep reinforcement learning bolus calculator integrated into a FAID system achieved 71.2% TIR, 0.9% TBR, and 26.7% TAR, performing competitively against a hybrid system using standard bolus calculators with CHO misestimation [46].

Several studies compared CL systems with and without automated meal detection. A basal-bolus CL controller In [14] improved TIR by 17%, reduced TAR by 25%, and lowered TBR by 1% compared to a system with detection, under scenarios with 50% unannounced meals. An online-tuned compound controller [23] outperformed an equivalent system without meal detection, improving TIR by 3.2% and reducing TAR and TBR by 3.0% and 0.1%, respectively. A hybrid CL system [12] with automated meal detection achieved a 5.3% increase in TIR and reduced TAR and TBR by 4.3% and 1.0% compared to a conventional CL system. In [9], integrating meal detection into a CL improved TIR by 3.9% and reduced TAR by 4.2%, while TBR increased slightly by 0.1%, compared to the same system without detection. In [36], adding a meal detector in a no-announcement scenario improved TIR by 19.9%, reduced TAR by 15.1%, and increased TBR by 0.7% compared to the baseline CL system. Finally, in [49], a CL system using moving horizon state estimator improved TIR by 13% over one using a Luenberger observer, due to faster meal response.

Several studies compared FAID systems against OL control systems. In [11], an ensemble meal detection approach improved TIR by 16.0% and 10.5% across two in-silico cohorts, reduced TAR by 11.9% and 5.2%, but increased TBR by 2.7% and 0.4%, respectively. Similarly, [16] reported an 18.4% improvement in TIR and a 19.3% reduction in TAR, with a modest 0.9% rise in TBR compared to OL system. Results from other studies comparing FAID with automated detection to systems relying on ideally announced meals are summarized in S2 Table.

Although many studies describe either algorithmic performance or glycemic outcomes, relatively few provide both. This limits the ability to judge whether high detection accuracy translates into meaningful improvements in glucose control. To address this gap, Table 2 compiles the subset of studies that report meal detection metrics alongside glycemic outcomes. Presenting these data together allows a direct view of how technical performance aligns with clinical benefit, across both in-silico and in-vivo evaluations. To synthesize these observations and provide a structured interpretation of how detection metrics translate into clinical outcomes, a structured framework-based analysis is presented in the Discussion.

**Table 2:** Meal detection performance and glycemic impact of fully automated insulin delivery systems.

Ref – [Year]	Method	Dataset (N)	Meal detection metrics	Glycemic outcomes
Daniels et al. [9] – [2022]	Machine Learning	In-silico [10]	Sens 76%, Pre 93%, F1-score 84, DT 38 min	**FAID:** TBR 1.4%, TIR 77.8%, TAR 20.7% vs **Hybrid CL:** TBR 1.5%, TIR 84.7%, TAR 13.7%
Mosquera-Lopez et al. [10] – [2023]	Machine Learning	In-vivo [15]	Sens 83%, FP/Day: 1.0, DT 25.9 min	Improved TIR by 9.1%, reduced TAR 10.8%, stable TBR vs Hybrid CL
Ibrahim et al. [11] – [2024]	Machine Learning	In-silico (20 + 47)	Sens 74.5-95%, Pre 83.5-96%, F1-score 82-88.5, FP/day 0.05-0.31, DT 21–31 min	***UVA*** **FAID:** TBR 1.53%, TIR 68.16%, TAR 13.59% **OL:** TBR 1.17%, TIR 57.69%, TAR 17.50% **Hovorka** **FAID:** TBR 5.79%, TIR 52.7%, TAR 14.99% **OL:** TBR 3.1%, TIR 36.67%, TAR 26.84%
Xu et al. [12] – [2021]	Control	In-silico [30]	Sens 100%, Pre 97.3%, F1-Score 98.6%, FP/Day 0.5, DT ≤ 60 min, 36 TP, 1 FP	**FAID:** TBR 0%, TIR 96.6%, TAR 3.3% vs **Hybrid CL:** TBR 1%, TIR 91.3%, TAR 7.6%
Mahmoudi et al. [13] – [2018]	Control	In-silico [9]	Sens 80%, Pre 100%, F1-Score 88.9%, DT 40 min, 36 TP, 1 FP, 2 FN	**FAID:** TBR 0%, TIR 71.6%, TAR 28.4% vs **Hybrid CL:** TBR 0%, TIR 85.6%, TAR 14.4%
Xie et al. [14] – [2017]	Control	In-silico [30]	Sens 76%, Pre 84%, F1-Score 80%, DT 45 min, FP/Day 0.44	**FAID:** TBR 11%, TIR 73%, TAR 23% vs **Hybrid CL:** TBR 5%, TIR 85.5%, TAR 9.5%
Pimentel et al. [17] – [2020]	Heuristic	In-silico [1]	DT 30–45 min	**FAID:** TBR 0%, TIR 90.7%, TAR 9.3% vs **Hybrid CL:** TBR 0%, TIR 91.4%, TAR 8.6%
Lee et al. [19] – [2008]	Heuristic	In-silico [1]	DT 30–40 min	**FAID:** MAD 38.6 g, Mean CGM 137 mg/dL vs **Hybrid CL:** MAD 91 g, Mean CGM 191 mg/dL
Mahmoudi et al. [20] – [2019]	Control	In-silico [9]	Sens 93%, Pre 100%, F1-Score 96.6%, 42 TP, 3 FN, DT 40 min	**FAID:** TBR 0%, TIR 83%, TAR 16%, vs **Hybrid CL:** TBR 0%, TIR 91%, TAR 9%
Harvey et al. [22] – [2014]	Heuristic	In-silico [10] +In-vivo [10]	Sens 88%, FP/day 0.1, DT 39 min	**FAID:** TBR 0%, TIR 74%, TAR 29.7% vs **Hybrid CL:** TBR 0%, TIR 89%, TAR 9.9%
Atlas et al. [27] – [2010]	Heuristic	In-vivo [7]	DT 23 min	**FAID:** TBR 0%, TIR 73%, TAR 27%
Dovc et al. [28] – [2020]	Heuristic	In-vivo [20]	DT 30.1-38.4 min	**FAID** (*faster insulin*): TIR 53.8% vs **FAID** (*standard insulin***):** TIR 58.6%
Samadi et al. [29] – [2017]	Heuristic	In-silico [30]	Sens 91.3%, Pre 90.7%, F1-Score 91%, TP 1370, FP 140, FN 130, FP/Day 0.47, MAE 23.1%	**FAID:** TBR 3.1%, TIR 76.8%, TAR 20% vs **Hybrid CL:** TBR 4.1%, TIR 85.4%, TAR 10.4%
Sayyar et al. [46] – [2024]	Machine Learning	In-silico [67]	Sens 64.3%, Pre 89.9%, F1-Score 74.93%, FP/Day 0.29	**FAID:** TBR 0.9%, TIR 71.2%, TAR 30.8% vs **Hybrid CL:** TBR 0.1%, TIR 76.2%, TAR 23.0%
Sala-Mira et al. [51] – [2019]	Control	In-silico [30]	Sens 90.5%, Pre 97.4%, F1-Score 93.8%, FP/day 0.1, 38 TP, 1 FP, 4 FN, DT 30 min	**FAID:** TBR 0%, TIR 78.9%, TAR 23.2% vs **Hybrid CL:** TBR 0%, TIR 86.9%, TAR 13%
Garcia-Tirado et al. [55] – [2021]	Control	In-silico [100]	DT 10–15 min	**FAID:** TBR 0%, TIR 76.1%, TAR 23.8%
Carlos et al. [69] – [2022]	Control	In-silico [10]	Sens 66.6%, Pre 81.5%, F1-Score 73.1%, FP/Day 0.6, DT 37.6 min, 5.3 TP, 2.7 FN, 1.2 FP	**FAID:** TBR 0%, TIR 94.1%, TAR 5.6% vs **Hybrid CL:** TBR 0%, TIR 90.9%, TAR 9.1%
Fathi et al. [74] – [2019]	Control	In-vivo [4]	Sens 100%, 0 FP, DT 35 min	**Area under the curve:** **FAID:** 18.0 ± 2.7 mmol/L vs **Hybrid CL:** 24.8 ± 11.5 mmol/L
Majdpour et al. [75] – [2021]	Control	In-vivo [9]	DT 30–40 min	**FAID** (*insulin-only*): TBR 4.7%, TIR 83.3%, TAR 12% vs **FAID** (*multihormone*): TBR 0.93%, TIR 81%, TAR 18%
Fushimi et al. [76]- [77] – [2019]	Control	In-silico [10]	DT 50–60 min	**FAID:** TBR 0%, TIR 85%, TAR 14.8% vs **Hybrid CL:** TBR 0%, TIR 83.3%, TAR 16.6%

N – number of patients; OL – open-loop system; CL – closed-loop system; FAID – fully closed-loop automated insulin delivery system; TIR – time-in-range (70-–180 mg/dL); TBR – time-below-range (<70 mg/dL); TAR – time-above-range (>180 mg/dL); CGM – continuous glucose monitoring; CHO – carbohydrates; Sens – sensitivity; Pre – precision; TP – true positive; FP – false positive; FN – false negative; DT – detection time; MAE – mean absolute error; MAD – mean absolute deviation. Only studies reporting both meal detection performance and glycemic outcomes were included here; extended tables are provided in the Supplementary Material (S1 Table and S2 Table). FP values are reported as presented in the original studies; for cross-study comparison, FP/day was used as the primary normalized metric when reported or derivable.

Table 2 focuses on the subset of studies that report both meal detection performance and glycemic outcomes, enabling direct assessment of how detection metrics translate into clinical impact relative to the comparator used in each study. Across these studies, high sensitivity does not consistently correspond to improved TIR or reduced TAR. Several in-silico evaluations report high sensitivity with moderate DT, yet observe lower TIR and higher TAR under FAID than under announced-meal hybrid CL [9,13,20,51], indicating that accurate detection alone cannot fully compensate for the absence of anticipatory pre-meal bolusing.

DT appears more closely related to postprandial hyperglycemia than to overall glycemic stability. Across Table 2, studies reporting longer DT, typically in the range of 30–45 min, generally maintain low TBR but exhibit higher TAR under FAID, particularly when compared with announced-meal hybrid CL [9,13,17,22,51]. This pattern suggests that delayed detection primarily affects the magnitude and duration of postprandial hyperglycemia rather than hypoglycemia risk, consistent with the delayed onset of insulin action following detection.

FP burden is more strongly linked to safety than to efficacy. Across Table 2, TBR remains consistently low in most FAID configurations, indicating that insulin delivery strategies are generally conservative. However, higher sensitivity or more aggressive detection does not systematically translate into improved TIR and may increase hypoglycemia risk if false detections are not constrained by insulin-on-board awareness and safety limits [11]. These results highlight that FP or FP/day should be interpreted jointly with the insulin compensation strategy rather than as isolated performance metrics.

The comparator system strongly influences the apparent benefit of automated meal detection. When FAID is compared against OL or non-automated baselines, clearer improvements in TIR and reductions in TAR are observed, even with moderate sensitivity and DT, as reported by [11]. In contrast, when FAID is compared against hybrid CL systems, outcomes are more heterogeneous. Some studies report improved TIR or reduced TAR under FAID relative to hybrid CL [12,69], whereas others show comparable or inferior performance compared with announced-meal hybrid CL [9,13,20,51]. These findings indicate that the relative advantage of automated meal detection depends strongly on the reference system used for comparison.

Differences in evaluation cohort further modulate these relationships. Notably, [11] evaluated the same detection framework on both the UVA/Padova and Hovorka simulators and reported different TIR and TAR outcomes across cohorts despite comparable detection performance, underscoring that the translation of detection metrics into glycemic benefit depends on cohort-specific physiological dynamics and model assumptions.

To place these study-level observations in a broader context, Fig 6 summarizes aggregate glycemic outcomes across in-silico and in-vivo evaluations. While in-silico studies generally report higher TIR and lower TAR under controlled conditions, in-vivo studies exhibit greater variability, particularly in TAR, reflecting physiological and behavioral heterogeneity. Across both settings, TBR remains consistently low, indicating that hypoglycemia is generally well managed. Together, Table 2 and Fig 6 illustrate that the clinical impact of automated meal detection depends not only on detection accuracy or timeliness, but also on the evaluation setting, comparator system, and integration of detection outputs within the insulin dosing strategy.

A variety of input data have been used for automated meal detection algorithms. CGM and insulin records are the most common, with 35 studies using CGM alone and 21 combining it with insulin data. Thirteen studies incorporated additional variables such as physical activity, meal timing, accelerometer or gyroscope signals, and even bowel sounds. Evaluation settings also varied: 38 studies relied on in-silico datasets, primarily based on the Dalla Man model [83] (via the UVA/Padova T1D simulator) or the Hovorka model [84]; 24 used in-vivo datasets; and 7 combined both. Real-world datasets have also been explored, most notably the publicly available OhioT1DM dataset, which includes CGM, insulin, and self-reported meal data from 12 insulin pump users. Fig 7 summarizes the distribution of input data across the reviewed studies.

## 4. Discussion

All reviewed meal detection methods can be broadly grouped into heuristic, machine learning, and control theory–based approaches, which differ mainly in how detection logic is constructed and how variability is handled. Heuristic methods rely on explicit decision rules applied to features derived from CGM or model residuals, offering simplicity and low computational burden but often requiring patient-specific parameters that limit robustness. Machine learning approaches instead learn detection patterns directly from data, enabling adaptation to nonlinear dynamics and heterogeneous inputs, but at the cost of larger data requirements, labeling effort, and sometimes higher computational load. Control-based methods infer meals as disturbances within a dynamical glucose-insulin model, leveraging observers or predictive control to integrate detection with insulin delivery, but often requiring complex modeling, tuning, and validation. While each class presents distinct strengths and limitations, their reported performance differences are increasingly shaped by evaluation design and system integration choices rather than detection logic alone.

While the Results section reports detection performance and glycemic outcomes as presented in individual studies, interpreting these metrics jointly remains challenging due to heterogeneous evaluation designs and reporting practices. To address this, we propose a structured framework that links commonly reported technical performance dimensions to their expected clinical relevance in FAID systems. The framework, summarized in Table 3, organizes evaluation across complementary dimensions such as detectability, alarm burden, timeliness, actionability, and net glycemic impact. This structure is intended to support clinically grounded interpretation of detection metrics rather than to impose additional reporting requirements.

**Table 3:** Framework for joint interpretation of technical meal detection metrics and clinical outcomes in fully automated insulin delivery systems.

Dimension	What it answers	Typical metrics / evidence	What is “good”	Common Pitfalls	How it connects to clinical care (TIR/TAR/TBR)	What you should ask the study to report
**Event detectability**	“Does the algorithm find true events?”	Sensitivity/Recall; FN rate	High recall **without** large burden	Reporting only sensitivity hides false alarms and timing	Necessary precondition for benefit; does not guarantee improved outcomes	Sensitivity **and** clear event definition + thresholds
**Alarm burden / usability**	“How noisy is it?”	**FP/day**, precision/PPV, specificity; alerts/day	Low FP/day and / or high precision, stable across days/subjects	FP/day incomparable if alert suppression / refractory periods differ; precision depends on prevalence	High FP/day can cause alert fatigue; in closed-loop can lead to unnecessary actions which **may worsen TBR**	FP/day with exact definition (per-day window), refractory period, precision
**Timeliness**	“Is it early enough to matter?”	**DT** (detection time), **lead time**, time-to-alarm distribution	Detects early enough to enable corrective action (aligned with insulin action / intervention delay)	DT unclear: measured from meal start? peak? threshold crossing? Averaging hides long tails	Earlier detection enables earlier insulin which can reduce **TAR**, increase **TIR**; late DT may not help spikes	DT definition + distribution (median/IQR) + horizon/window
**Actionability / control relevance**	“Can the output be used safely to dose insulin?”	Decision logic, insulin constraints, safety layers (Insulin-on-board limits, max bolus, confidence gating)	Conservative safety layers + clearly defined control policy	Great detection metrics may be unsafe if control policy is aggressive; control not described	Safety layers strongly influence **TBR** risk; confidence gating reduces harm from false positives	Control policy description + safety constraints + how false positives are handled
**Clinical impact (net benefit)**	“Does it improve glucose outcomes?”	**TIR/TAR/TBR**, mean glucose, variability; Δ vs comparator	Improved TIR and/or reduced TAR **without** worsening TBR	Reporting TIR without stating ranges; mixing mg/dL vs mmol/L thresholds; short windows	This is what clinicians care about most; should be tied to comparator and duration	TIR/TAR/TBR with thresholds + Δ vs comparator + duration
**Comparator fairness**	“Is the baseline meaningful?”	OL vs hybrid CL vs standard CL; announced vs unannounced meals	Comparator matches real clinical use-case	Comparing to weak baseline inflates gains; different meal announcement assumptions	Determines whether reported improvements translate to practice	Clearly state comparator + meal announcement scenario
**Generalizability**	“Will it work outside this dataset?”	Free-living validation, external cohort, multi-site data	Evidence beyond in-silico; at least free-living or external test	In-silico success often overstates real-world performance	Real-world deployment depends on free-living robustness	Setting (in-silico/in-vivo/free-living) + external validation flag
**Validation integrity**	“Are results inflated by leakage?”	Subject-wise splits, nested CV, LOSO; leakage checks	**Subject-wise** evaluation; clear separation of training/testing	Record-wise splits in time-series CGM can leak subject patterns	Over-optimistic metrics will not translate clinically	Split type (subject-wise vs record-wise), CV scheme, tuning method
**Reproducibility**	“Can others verify/compare?”	Open data/code; standardized definitions	Open artifacts or at least detailed methods	Proprietary data + vague definitions blocks comparability	Improves credibility and accelerates translation	Dataset availability, code availability, complete metric definitions

Interpreting the reviewed studies through this framework reveals that detection metrics do not translate uniformly into clinical outcomes. High sensitivity alone does not consistently correspond to improved TIR, especially in studies reporting longer detection times. Detection timeliness appears more closely related to postprandial TAR, whereas false positives mainly influence safety outcomes, depending on the presence of insulin constraints and safety layers. The results summarized in Table 2 and Figs 4–6 therefore suggest that clinical impact depends on the joint interaction of detection behavior, control policy, and evaluation baseline. These interactions are shaped not only by algorithm design, but also by the choice of input data, evaluation setting, and deployment context.

In practice, several meal detection algorithms rely solely on CGM data, while others incorporate additional signals such as insulin delivery, physical activity, or accelerometer data. These approaches demonstrate technical feasibility, but the current evidence remains largely based on controlled or simulated evaluations, with limited real-world clinical validation, and should therefore be interpreted as encouraging early-stage evidence rather than established clinical readiness. A recent feasibility study demonstrated a real-world implementation of a FAID system employing a self-adapting algorithm in adults with type 1 and type 2 diabetes, without requiring manual meal announcements [85]. The system improved TIR and reduced hyperglycemia, supporting the clinical feasibility of managing unannounced meals. While the algorithm itself was not described in detail, the study illustrates the practical potential of such systems and highlights the need for broadly generalizable solutions. Successfully integrating these methods into wearable, low-power, and user-friendly devices will require addressing challenges related to sensor reliability, data synchronization, computational efficiency, and clinical robustness. Continued advances in sensor and algorithm design suggest that real-time, multi-sensor meal detection could become a practical component of future FAID systems, pending further development and clinical validation.

From a safety perspective, FP represent one of the most critical considerations in FAID systems. FP are of particular concern because they can lead to inappropriate insulin dosing and increase hypoglycemia risk. Minimizing FP without substantially compromising sensitivity or DT therefore remains a key challenge. In practice, detection performance must be interpreted as a trade-off among sensitivity, FP, and DT rather than optimized for a single metric. One factor contributing to elevated FP rates is signal quality. Noisy CGM signals or irregular inputs can mislead detection algorithms and result in spurious detections. Applying smoothing filters or other signal preprocessing techniques can help mitigate these effects and improve detection reliability.

Timeliness represents a second critical dimension, reflecting the unavoidable physiological delay between meal ingestion and observable glucose response. Meal detection algorithms face a time-delay challenge, as most rely on identifying deviations between predicted and actual BG levels, a process that inherently takes time. The physiological delay between meal intake and its observable effect on BG typically ranges from 15 to 30 minutes. As a result, many algorithms detect meals between 25 and 60 minutes after ingestion, as reported in the reviewed studies. At these time scales, detection is generally more effective for attenuating the magnitude and duration of postprandial hyperglycemia than for preventing glucose excursions altogether. Such detection delays can lead to suboptimal insulin delivery and increase the risk of both postprandial hyperglycemia and hypoglycemia. Reducing this latency is essential for the development of future FAID systems. Advances in CGM technology are helping to reduce sensor lag and enable earlier detection of glucose excursions [86]. Similarly, faster-acting insulin formulations shorten the time required to mitigate post-meal glycemic rises [87]. Sophisticated algorithms incorporating predictive modelling and machine learning may enhance early meal detection and enable more timely insulin adjustments. Nevertheless, extensive clinical validation remains necessary to ensure the safety and effectiveness of these approaches in real-world settings.

Imbalanced data is another challenge, as meal events occur less frequently than non-meal periods, leading to skewed class distributions that can bias detection models. This issue is compounded by real-world confounders such as rescue CHO intake and exercise, where glucose excursions unrelated to meals may be misclassified and trigger inappropriate insulin delivery. Data augmentation and scenario-aware evaluation can partially address imbalance, but future studies should explicitly test robustness under such confounding conditions. Accurate estimation of meal size following detection is also critical, as overestimation may lead to excessive insulin dosing and increase hypoglycemia risk, particularly in the absence of user confirmation.

These algorithmic and physiological constraints place strong demands on how performance is evaluated and reported, particularly given the predominance of in-silico evaluations, substantial heterogeneity in validation methodologies and system configurations, the still limited number of real-world clinical evaluations, and the lack of standardized definitions and reporting practices across studies. Across the reviewed studies, key performance metrics are not consistently defined or reported, and several publications omit one or more commonly used measures altogether. Reported performance metrics cannot be interpreted independently of evaluation design, as differences in data partitioning and validation strategies, including retrospective versus prospective evaluation, introduce systematic heterogeneity across studies, as summarized in S3 Table and S4 Table. This heterogeneity limits direct quantitative comparison between approaches and complicates the interpretation of reported trade-offs. Additionally, no formal risk-of-bias or certainty-of-evidence assessment was conducted because the reviewed literature consisted predominantly of algorithm-development, simulation, and engineering studies for which standard clinical risk-of-bias tools were not directly applicable. This should be considered a limitation of the review. To facilitate reproducibility and meaningful benchmarking, future studies would benefit from adopting a minimal, standardized set of performance metrics capturing complementary aspects such as detection accuracy, detection latency, and robustness to false detections, alongside clearly defined evaluation protocols and validation settings.

This effect is also evident when contrasting in-silico and in-vivo evaluations. Fig 4 shows that in-vivo studies tend to report lower FP rates and shorter DT than in-silico studies. These apparent differences should be interpreted in the context of evaluation design rather than as evidence of intrinsically superior in-vivo algorithm performance. In-vivo studies are fewer in number and are typically conducted under controlled clinical protocols with conservative algorithm tuning and limited observation windows, which can reduce the opportunity for false detections and extreme delays to occur. In contrast, in-silico studies often evaluate detection algorithms across larger virtual cohorts, diverse meal patterns, and stress scenarios, leading to more comprehensive reporting of FP and DT. Importantly, even low FP rates in in-vivo settings remain clinically relevant, as false meal detections can trigger inappropriate insulin delivery and pose safety risks, underscoring the need for continued FP reduction and broader in-vivo validation.

Alongside these challenges, several recent approaches have explored macronutrient and carbohydrate estimation as a means of reducing reliance on manual meal announcement [88,89]. However, the benefit of such approaches depends critically on how the estimated information is incorporated into insulin delivery. In unannounced meal scenarios, meal detection typically occurs with a delay relative to ingestion, at which point glucose dynamics already reflect both meal absorption and insulin on board. Using macronutrient estimates to trigger large corrective insulin doses at this stage may increase the risk of late postprandial hypoglycemia. In contrast, several approaches treat macronutrient estimates as auxiliary information to constrain, modulate, or gradually adjust insulin delivery rather than as a direct substitute for pre-meal bolusing. This distinction suggests that macronutrient estimation may complement meal detection only when integrated conservatively within the control strategy, while its naive use does not eliminate and may even exacerbate the challenges associated with delayed meal detection.

From an algorithmic perspective, most existing methods rely on physiological models to estimate the patient’s state, predict future glucose levels, and compare them with measured values. However, such models often fall short in accurately capturing the physiological variability of individuals with T1D. In recent years, data-driven approaches have gained popularity due to their ability to learn complex patterns directly from data. Nonetheless, training these models requires large datasets, and collecting data with numerous unannounced meal events is impractical due to safety concerns. Recent studies have reported promising results in generating individualized synthetic data using generative adversarial networks [90–92]. A variant known as conditional generative adversarial networks enables data generation conditioned on specific inputs, such as insulin delivery and CHO intake [93]. These models offer a potential solution for augmenting training data with realistic and scenario-specific samples.

While prior work has demonstrated a wide range of meal detection paradigms, the reviewed literature reveals clear and recurring trade-offs across methodological families. Heuristic and signal-processing approaches offer transparency, low computational complexity, and predictable behavior, but their performance can be brittle across individuals and under free-living confounders such as exercise or rescue carbohydrate intake. Machine learning and deep learning methods improve pattern recognition and reduce reliance on manual feature design, yet often lack external or free-living validation and are sensitive to subject-level variability when evaluation is not subject-wise. Control-oriented approaches most directly target clinical outcomes by integrating detection with insulin delivery, but their effectiveness depends strongly on modeling assumptions, safety constraints, and controller design. Taken together, these observations motivate a forward research agenda that prioritizes free-living external validation, standardized definitions for metrics such as DT and FP/day, harmonized evaluation procedures, and reporting practices that explicitly link technical detection performance to clinical outcomes such as TIR, TBR, and TAR.

### Author perspective and future research priorities

Based on the evidence synthesized in this review, we outline below our perspective on the directions most likely to deliver meaningful clinical impact in automated meal detection for FAID systems.

- From detection metrics to system-level glycemic benefit.

Future progress should move beyond optimizing standalone detection metrics. While sensitivity, FP, and DT are necessary to characterize algorithm behavior, they are insufficient endpoints. Across reviewed studies, similar detection performance often leads to widely different glycemic outcomes, indicating that detection itself is no longer the dominant bottleneck. The primary objective should be net glycemic benefit, namely improvements in TIR with reductions in TAR while preserving low TBR after insulin compensation. In this context, data-driven detection should be prioritized over purely physiological modeling, as it better captures inter- and intra-subject variability under unannounced meals, stress, and exercise. Moreover, physiological delays in glucose appearance impose a lower bound on actionable detection, meaning that marginal reductions in DT alone are unlikely to yield proportional clinical benefit without faster insulin pharmacokinetics or alternative sensing.

- Robustness, safety, and prospective validation over idealized performance.

Retrospective and in-silico evaluations remain useful for early development, but retrospective-only results are increasingly insufficient to support clinical relevance. Future work should prioritize prospective validation under realistic conditions, where confounders such as exercise, rescue carbohydrate intake, and sensor artifacts dominate performance. In FAID systems, false positives are particularly critical because they can trigger inappropriate insulin delivery. Given unavoidable detection delays, large corrective boluses after detection increase hypoglycemia risk. Algorithms for unannounced meals should therefore emphasize low FP operation, robustness under confounders, and conservative insulin modulation strategies such as micro-bolusing or adaptive basal adjustments. More aggressive corrections may only become viable with faster insulin analogs and explicit safety layers.

- Fair comparison, uncertainty-aware detection, and human-in-the-loop design.

Reported performance differences often reflect different operating points along the sensitivity-FP-DT trade-off rather than intrinsic methodological superiority. Fair comparison therefore requires benchmarking algorithms under fixed operating constraints, such as predefined FP limits. In addition, detection outputs should move beyond binary triggers toward uncertainty-aware representations that propagate confidence to the control layer and enable graded insulin responses. Human-in-the-loop strategies remain underexplored and should be treated as a controllable system component rather than a fallback. Confidence-dependent alerts, selective confirmation, and adaptive safety thresholds offer practical mechanisms to balance automation and safety under uncertainty.

Together, these priorities point toward a shift from standalone detection optimization to system-level integration, safety-aware insulin compensation, robustness to real-world variability, and prospective validation.

In summary, while significant progress has been made in developing algorithms for automated meal detection, several critical challenges remain before these methods can be reliably integrated into real-world FAID systems. Key issues include reducing detection delay, minimizing FPs, ensuring robustness across diverse scenarios, and achieving accurate meal size estimation. Advances in sensor technology, algorithm design, and synthetic data generation offer promising opportunities to address these limitations. Continued interdisciplinary collaboration, along with rigorous clinical validation, will be essential to translate these innovations into safe and effective FAID systems.

## 5. Conclusion

Automated meal detection remains an active and evolving area of research aimed at advancing toward FAID systems. Recent developments have introduced innovative algorithms, particularly those based on machine learning and control theory, that can detect unannounced meals with improving accuracy by utilizing CGM signals and other physiological data. However, major limitations persist. The inherent physiological delay between food intake and its impact on blood glucose, the lag in CGM sensing, and the delay between insulin administration and its ability to reduce blood glucose collectively limit the effectiveness of these systems in real-world conditions. Although some studies report improvements in glycemic outcomes such as increased TIR and reduced TAR and TBR compared to basic CL systems without automated meal detection and compensation, these solutions still do not match the precision of pre-meal insulin dosing. Furthermore, issues like false positives, inaccurate meal size estimation, and poor generalization to daily-life variables such as exercise, stress, and rescue CHO intake continue to challenge their robustness. Looking ahead, further progress in automated meal detection is unlikely to be driven by improvements in detection accuracy alone. Instead, future work should emphasize system-level integration, including robust insulin modulation strategies, improved handling of daily-life confounders, and prospective validation in free-living settings. In this context, human-in-the-loop designs remain comparatively underexplored and warrant greater attention. Carefully designed human-in-the-loop mechanisms, such as confidence-aware alerts, selective user confirmation, or adaptive safety constraints, may help balance automation with safety, particularly under uncertainty. When combined with advances in sensing technologies and insulin pharmacokinetics, such approaches provide a promising pathway toward clinically robust and low-burden FAID systems.

## Supporting information

S1 Checklist PRISMA checklist. From: Page MJ, McKenzie JE, Bossuyt PM, Boutron I, Hoffmann TC, Mulrow CD, et al. The PRISMA 2020 statement: an updated guideline for reporting systematic reviews. BMJ 2021;372:n71. https://doi.org/10.1136/bmj.n71. This work is licensed under CC BY 4.0. To view a copy of this license, visit https://creativecommons.org/licenses/by/4.0/.
(PDF)

S1 Table Performance metrics comparison for unannounced meal detection techniques. (DOCX)

S2 Table Glycemic outcomes with automated meal detection vs manual or no meal announcements. (DOCX)

S3 Table Data partitioning, validation, and performance reporting in machine learning-based meal detection studies. (DOCX)

S4 Table Overview of automated meal detection studies and reported outcomes. (DOCX)

S1 Fig Distribution of reported performance metrics across automated meal detection method categories. In each box plot, the orange horizontal line indicates the median, the green triangle indicates the mean, and the black circles represent outliers. Boxes show the interquartile range, and whiskers indicate the spread of non-outlier values. (DOCX)
