# Supplementary material for: On the road to fully automated insulin delivery: A systematic review of meal announcement free algorithms
Source: PLOS Digit Health. 2026 Jul 9;5(7):e0001492. doi: 10.1371/journal.pdig.0001492 (PMC13349122; doi:10.1371/journal.pdig.0001492)
Supplement: S1 Table — (DOCX) [file pdig.0001492.s002.docx]

**S1 Table. Performance metrics comparison for unannounced meal detection techniques**

| **Ref – [Year]** | **Dataset** | **N** | **Days** | **Data Used** | **Validation Setting** | **Therapy** | **Category** | **Method** | **Performance Metrics** |
| --- | --- | --- | --- | --- | --- | --- | --- | --- | --- |
| Dassau et al. (7) – [2008] | In-vivo | 17 | - | CGM | Retrospective | Insulin-only | Heuristic approach | Backward difference (BD), Kalman filter (KF), BD + KF, and 2^nd^ derivative of glucose | *two-out-of-three:* DT: 30 min, ΔG: 15 ± 10 mg/dL  *three-out-of-four_:_* DT: 32 min, ΔG: 21 ± 9 mg/dL  ∴ΔG (BG at detection – preprandial) |
| Ramkissoon et al. (8) – [2018] | In-silico  (UVA/Padova) | 10 | 500 | CGM, insulin | Retrospective | Insulin-only | Control system theory | Unscented-KF (UKF) along with decision rules | *Highest sensitivity:*  Sens: 92%, TP: 1374, FP: 716, FN: 126, FP/day: 1, DT: 25 min  *Trade-off:*  Sens: 83%, TP: 1242, FP: 96, FN: 259, FP/day: 0, DT: 35 min  *Low False Positive:*  Sens: 52%, TP: 776, FP: 10, FN: 724, FP/day: 0, DT: 40 min |
| Daniels et al. (9) – [2022] | In-silico  (UVA/Padova) | 10 | 60 | CGM, insulin | Retrospective | Insulin-only | Machine learning approach | LSTM network-based seq2seq model | Sens: 76, Pre: 93, F1-Score: 84, DT: 38 min |
| Mosquera- Lopez et al. (10) – [2023] | In-silico  (UVA/Padova & open-source simulator by OHSU);  In-vivo | 199;  13 | 14;  0.167 (4 hours) | CGM, insulin, time | Prospective | Insulin-only | Machine learning approach | Multioutput neural network | *In-silico validation:*  Sens: 79 to 90%, DT: 27.5 ± 4.8 min  *With real T1D subjects:*  Sens: 83.3%, FP/Day: 1.0, DT: 25.9 ± 0.9 min |
| Ibrahim et al. (11) – [2024] | In-silico  (UVA/Padova and Hovorka);  In-vivo | 20, 47;  12 | 30;  30;  349 | CGM, insulin | Retrospective | Insulin-only | Machine learning approach | Ensemble machine learning approach combining NN, LR, and RF | *In-silico (average):*  *AND:* Sens: 74.5, Pre: 96, F1-Score: 82, FP/Day: 0.05, DT: 31 min  *Voting:* Sens: 85.5, Pre: 92.5, F1-Score: 88, FP/Day: 0.12, DT: 25 min  *OR:* Sens: 95, Pre: 83.5, F1-Score: 88.5, FP/Day: 0.31, DT: 21 min  *In-vivo (OhioT1DM):*  *AND:* Sens: 47, Pre: 83, F1-Score: 60, FP/Day: 0.07, DT: 43 min  *Voting:* Sens: 61, Pre: 72, F1-Score: 66, FP/Day: 0.17, DT: 41 min  *OR:* Sens: 71, Pre: 62, F1-Score: 66, FP/Day: 0.32, DT: 33 min |
| Xu et al. (12) – [2021] | In-silico  (UVA/Padova) | 30 | 2 | CGM | Retrospective | Insulin-only | Control system theory | UKF with decision rules | Sens: 100%, Pre: 97.3%, F1-Score : 98.6%, FP/Day: 0.5, DT: ≤ 60 min, TP: 36, FN: 0, FP: 1 |
| Mahmoudi et al. (13) – [2018] | In-silico  (MVP model) | 9 | 1.5 | CGM, insulin | Retrospective | Insulin-only | Control system theory | KF with CUSUM and threshold tests | DT: 40 min, Sens: 80%, Pre : 100%, F1-Score : 88.9%, TP: 36, FN: 9, FP: 0 |
| Xie et al. (14) – [2017] | In-silico  (UVA/Padova) | 30 | 2 | CGM, insulin | Retrospective | Insulin-only | Control system theory | Variable state dimension using KF | Sens: 76%, Pre: 84%, F1-Score: 80%, DT: 45 min, FP/Day: 0.44 |
| Pimentel et al. (17) – [2020] | In-silico  (UVA/Padova) | 1 | 1.5 (36 hours) | CGM, insulin | Retrospective | Insulin-only | Heuristic approach | Feedback scheme | DT: 30-45 min |
| Lee et al. (18) – [2009] | In-silico  (UVA/Padova) | 200 | 1.5 (36 hours) | CGM | Retrospective | Insulin-only | Heuristic approach | KF along with decision rules based threshold test | Sens: 80%, Pre: 92.4%, F1-Score: 86.9%, TP: 656, FP: 54, FN: 144, FP/Day: 0.18, DT: 31 min, meal estimation error: 36.41 g |
| Lee et al. (19) – [2008] | In-silico  (Hovorka) | 1 | 3 | CGM | Retrospective | Insulin-only | Heuristic approach | KF and rate-of-change of glucose concentration | DT: 30-40 min |
| Mahmoudi et al. (20) – [2019] | In-silico  (Medtronic virtual patient - MVP) | 9 | 1.5 | CGM, insulin | Retrospective | Insulin-only | Control system theory | KF along with CUSUM and threshold tests | Sens: 93%, Pre: 100%, F1-Score: 96.6%, TP: 42, FN: 3, DT: 40 min  26 meals were underestimated and 16 were overestimated with median bias of -19 g and 21 g, respectively. |
| Kolle et al. (21) – [2020] | In-vivo | 12 | - | CGM | Retrospective | Insulin-only | Heuristic approach | LDA for CGM and R_a_, GRID algorithm, and threshold tests | Four methods were used: *LDA R_a_*, *LDA CGM, Threshold-based, GRID*  *Best result achieved with LDA CGM:*  Sens: 90%, FP/day: 1.37, DT: 11.78 min. |
| Harvey et al. (22) – [2014] | In-silico  (UVA/Padova);  In-vivo | 10;  12 | 1;  1 | CGM | Prospective | Insulin-only | Heuristic approach | GRID algorithm | *In-silico:*  Sens: 88%, FP/day: 0.1, DT: 39 min *In-vivo:* Sens: 87.5%, FP/day: 1.6, DT: 42 min |
| Zheng et al. (25) – [2019] | In-silico  (UVA/Padova);  In-vivo | 100;  15 | 1;  4 | CGM, insulin, HR | Retrospective | Insulin-only | Heuristic approach | Euclidean distance between predicted and measured glucose | *In-silico results*  Sens: 88%, Pre: 93.3%, F1-Score: 90.6%, DT: 25.7 min, meal size error: 1.2 g  *Clinical data*  TP: 23, FP: 7, Sens: 76.7%, FP/Day: 0.11 |
| Weimer et al. (26) – [2016] | In-vivo | 61 | 17 | CGM | Retrospective | Insulin-only | Heuristic approach | Physiological parameter-invariant (PAIN) based meal detector | PAIN meal detector results  Sens: 86.9% with FP/day: 2.01 |
| Atlas et al. (27) – [2010] | In-vivo | 7 | 1.33 (32 hours) | CGM, insulin | Prospective | Insulin-only | Heuristic approach | FL | Average DT: 23 min |
| Dovc et al. (28) – [2020] | In-vivo | 20 | 1.125 (27 hours) | CGM, insulin | Prospective | Insulin-only | Heuristic approach | FL | DT: 30.1 - 38.4 min |
| Samadi et al. (29) – [2017] | In-silico  (UVA/Padova) | 30 | 10 | CGM, insulin | Retrospective | Insulin-only | Heuristic approach | Fuzzy logic (FL) | *Average results:*  Sens: 91.3%, Pre: 90.7%, F1-Score:91%, TP: 1370, FP: 140, FN: 130, FP/Day: 0.47, MAE: 23.1% (meal-size estimation error) |
| Samadi et al. (30) – [2018] | In-vivo | 11 | 2.57 (60 hours) | CGM | Prospective | Insulin-only | Heuristic approach | FL | *Overall results (meals + snacks):*  Sens: 88%, Pre: 79%, F1-Score: 83.4%, TP: 103, FN: 14, FP: 27, FP/Day: 1.05, DT: 34.8 min, Meal size estimation of MAD: 21.2 g  Sens(meals): 93.5%, Sens(snacks): 68% |
| Ornetzeder et al. (32) – [2019] | In-vivo | 10 | 5 | CGM | Retrospective | Insulin-only | Heuristic approach | UKF, FL, and GRID algorithm ((8,22,29)) | *Average result T1DM subjects:*  Harvey (22): Sens: 77%, FP/Day: 0.6, DT: 19.1 min  Samadi (29): Sens: 73.7%, FP/Day: 0.9, DT: 12.7 min  Ramkissoon (8): Sens: 75%, FP/Day: 0.4, DT: 19.6 min |
| Maria et al. (34) – [2021] | In-silico  (UVA/Padova) | 30 | 2.1 | CGM | Retrospective | Insulin-only | Machine learning approach | Ensemble methods using LSTM | *Average:* Sens: 74.31%, Spe: 69.38%, Acc: 70.56%, DT: 9.20 min  *Dynamic weighting:* Sens: 74.34%, Spe: 69.27%, Acc: 70.48%, DT: 9.71 min  *Voting:* Sens: 73.80%, Spe: 70.67%, Acc: 71.42%, DT: 9.16 min  *Min/Max:* Sens: 74.62%, Spe: 71.49%, Acc: 72.24%, DT: 8.95 min |
| Lu et al. (35) – [2024] | In-vivo | 82 | 10497 | Timestamped meal logs | Retrospective | Insulin-only | Machine learning approach | LSTM | *Best results (1-layer LSTM):*  Sens: 97.72% ; Pre: 93.51%; F1-Score: 95.33%; FP/Day: 0.25; FN/Day: 0.07 |
| Askari et al. (37) – [2022] | In-vivo | 11 | 4315 | CGM, insulin,  CHO, PA | Retrospective | Insulin-only | Machine learning approach | LSTM NN, LSTM-1D Conv, 2D ConvLSTM NN, and Bi-LSTM 1D Conv | Overall performance (average)  *LSTM NN:* Acc: 91.52%, Sens: 91.52%, Pre: 95.57%, F1-Score: 93.15%  *LSTM 1D Conv:* Acc: 92.32%, Sens: 92.32%, Pre: 95.94%, F1-Score: 93.68%  *2D ConvLSTM NN:* Acc: 88.64%, Sens: 88.64%, Pre: 95.25%, F1-Score: 91.40%  *Bi-LSTM 1D:* Acc: 92.29%, Sens: 92.29%, Pre: 96.17%, F1-Score: 94.00% |
| Fazakas et al. (38) – [2024] | In-vivo | 351 | 354 | Real-world wearable data (accelerometer & gyroscopes) | Retrospective | Insulin-only | Machine learning approach | LSTM | *Mean results:*  Sens: 98.8%; Pre: 99%; F1-Score: 98.9%  DT: 5 to 6 seconds |
| Zheng et al. (39) – [2020] | In-silico  (Hovorka) | 4 | 160 | CGM, insulin, CHO | Retrospective | Insulin-only | Machine learning approach | Extended Isolation Forest | *Partial meal announcement:* Sens: 90.8%, FP/Day: 0.39, DT: 39min  *No meal announcement:* Sens: 90%, DT: 35.1 min |
| Federico et al. (42) – [2023] | In-silico  (UVA/Padova) | 30 | 30 | CGM | Retrospective | Insulin-only | Machine learning approach | Decision tree, Random forest, SVM, Gaussian NB, Complement NB, Feedforward NN, LSTM NN, Threshold | *Average results of all models used:*  Sens: 73.6%; Pre: 70%; F1-Score: 69.3%  The paper identifies different best models by cohort:  *Feedforward NN* (adults: Sens 62.1%, Pre 86.8%, F1-Score 72.2%), *SVM* (adolescents: Sens 90.3%, Pre 72.7%, F1-Score 80.5%), and *Threshold* (children: Sens 61.4%, Pre 93.8%, F1-Score 74.3%) |
| Kolle et al. (43) – [2019] | In-vivo | 11 | - | Bowel sounds | Retrospective | Insulin-only | Machine learning approach | SVM | Sens: 40-50, FP/Day: 0.08, DT: 10 min |
| Carvalho et al. (44) – [2023] | In-vivo (OhioT1DM) | 12 | - | CGM | Retrospective | Insulin-only | Machine learning approach | RF, decision tree, MLP, AdaBoost, Gradient boosting, Gaussian NB | *Best results (participant 544)*  *RF:*  Sens: 91; Pre: 76; F1-Score: 83  *Average results:*  Sens: 59; Pre: 45; F1-Score: 50, FP: 19.2 |
| Sayyar et al. (46) – [2024] | In-silico (UVA/Padova) | 67 | 14 | CGM, insulin | Retrospective | Insulin-only | Machine learning approach | Deep reinforcement learning | Overall average results:  TP: 36, FP: 4.09, FN: 20, Sens: 64.3%, Pre: 89.8%, F1-Score: 74.93%, FP/Day: 0.29, and DT: 34.1 min  *Breakfast*  Sens: 57.7%, and DT: 37.9 min  *Lunch*  Sens: 95.2%, and DT: 35.0 min  *Snacks*  Sens: 8.3%, and DT: 29.2 min  *Dinner*  Sens: 95.8%, and DT: 34.4 min |
| Sala-Mira et al. (51) – [2019] | In-silico  (UVA/Padova) | 30 | 14 | CGM | Retrospective | Insulin-only | Control system theory | Super-twisting based residual generator | *Meal detector performance:*  TP: 38.0, FP: 1.0, FP/day: 0.1, FN: 4.0, Sens: 90.5, Pre: 97.4%, F1-Score: 93.8%, DT: 30.0 min  *Within AID system simulation***:**  Sens: 92.8, FP/day: 0.1, DT: 30.0 min |
| Faccioli et al. (53) – [2022] | In-vivo | 30 | 14 | CGM | Retrospective | Insulin-only | Control system theory | Super-twisting based residual generator and KF | Sens: 70%, Pre: 73%, F1-score: 68%, TP: 16, FN: 6, FP: 7, FP/day: 1.4, DT: 45 min |
| Garcia-Tirado et al. (55) – [2021] | In-silico  (UVA/Padova) | 100 | - | CGM, insulin | Retrospective | Insulin-only | Control system theory | Bolus priming system with KF & Model Predictive Control (MPC) | DT: 10-15 min |
| Turksoy et al. (64) – [2016] | In-vivo | 9 | 1.33 (32 hours) | CGM | Retrospective | Insulin-only | Control system theory | multivariable adaptive AID system using UKF | Sens: 96.8%, Pre: 98.4%, F1-Score: 97.6%, FP/Day: 0.08, TP: 63, FN: 2, FP: 1 |
| Ramkissoon et al. (66) – [2017] | In-silico  (UVA/Padova) | 10 | 10 | CGM | Retrospective | Insulin-only | Control system theory | cross-covariance  along with UKF | *without exercise:* Sens: 90%, Spe: 95%, Acc: 94%, DT: 28 min  *with exercise:* Sens: 88%, Spe: 95%, Acc: 94%, DT: 28 min |
| Godoy et al. (68) – [2021] | In-silico  (UVA/Padova);  In-vivo | 30;  11 | 1.5;  5 | CGM, insulin, CHO | Retrospective | Insulin-only | Control system theory | Feedback scheme-based algorithm | *In-silico results:*  TP: 163, FP: 1, FN: 2, Sens: 98.78%, Pre: 99.39%, F1-Score: 99.09%, FP/Day: 0.67, DT: 4.6 min, CHO estimation error: 14%  *In-vivo results:*  TP: 184, TN: 263, FP: 9, FN: 2, Sens: 98.91%, Pre: 95.34%, F1-Score: 97.09%, Spe: 97.48%, Acc: 98.10%, FP/Day: 0.16, DT: 9 min  CHO estimation error: 8.43% |
| Carlos et al. (69) – [2022] | In-silico  (UVA/Padova) | 10 | 2 | CGM, insulin, CHO | Retrospective | Insulin-only | Control system theory | KF with Feedback scheme-based algorithm | *Meal detection performance:*  TP: 5.3, FN: 2.7, FP: 1.2, Sens: 66.6%, Pre: 81.5%, F1-Score: 73.1%, FP/Day: 0.6, DT: 37.6 min, CHO estimation error: 51% |
| Mahmoudi et al. (70) – [2018] | In-silico  (MVP model) | 9 | 13.5 | CGM, insulin | Retrospective | Insulin-only | Control system theory | Moving horizon estimator (MHE) | Sens: 96%, Pre: 95.6%, F1-Score: 95.6%, FP/Day: 0.15, TP: 43, FP: 2, FN: 2, DT: 33 min |
| Chen et al. (71) – [2019] | In-silico  (Hovorka) | 10 | 30 | CGM | Retrospective | Insulin-only | Control system theory | moving horizon estimator | Sens: 88.5%, DT: 19.99 min, FP/Day: 2.6, CHO estimation Acc: 58.14% |
| Mahmoudi et al. (72) – [2017] | In-silico  (Hovorka + MVP) | 1 | 50 | CGM | Retrospective | Insulin-only | Control system theory | adaptive UKF | Sens: 99.5%, TP: 199, FN: 1, DT: 58.4 min |
| Meneghetti et al. (73) – [2020] | In-silico  (UVA/Padova) | 100 | 90 | CGM | Retrospective | Insulin-only | Control system theory | ARMAX model and KF | *Meal detection performance:*  Sens: 86.8%, FP/day: 0.15, DT: 56 min |
| Fathi et al. (74) – [2019] | In-silico  (Hovorka);  In-vivo | 512;  4 | 832;  0.375 (9 hours) | CGM, insulin | Prospective | Insulin-only | Control system theory | KF | *In-silico results (total meals: 1536)*;  Sens: 93.23%, Pre: 95.72%, F1-Score: 94.46%, FP/Day: 0.07, FP: 64, DT: 40 min  *In-vivo results (total meals: 12);*  Sens: 100%, FP: 0, DT: 35 min |
| Majdpour et al. (75) – [2021] | In-vivo | 9 | 1 | CGM, insulin | Prospective | Insulin, glucagon, pramlintide | Control system theory | KF with decision rule | DT: 30-40 min |
| Fushimi et al. (76) – [2019] | In-silico  (UVA/Padova);  In-vivo | 10;  5 | 1.5;  1.5 | CGM | Retrospective | Insulin-only | Control system theory | ARG algorithm with SSG and KF | *In-silico results:*  DT: 50-60 min  *In-vivo results:*  Sens: 83.3, FP/Day: 0.27, TP: 22, FP: 2, FN: 2, DT: 20.2 min |

*N – number of patients; Sens – sensitivity; Spe – specificity; Acc – accuracy; Pre – precision; DT – detection time; min – minutes; TP – true positive; TN – true negative; FP – false positive; FN – false negative; FP/day – false positives per day; CUSUM – cumulative sum; LSTM – long short-term memory; CGM – continuous glucose monitoring; CHO – carbohydrates intake information; HR – heart rate; SVM – support vector machine; NB – naïve bayes; NN – neural network; MLP – multi-layer perceptron; LR – logistic regression; RF – random forest; PA – physical activity information; LDA – linear discriminant analysis; R_a_ – rate of appearance; GRID – glucose rate increase detector; ARMAX – autoregressive moving-average model with exogenous inputs; ARG – automated regulation of glucose; SSG – switching signal generator; MAD – mean absolute deviation*

- *Citation numbers are consistent with those in the main manuscript.*
- *FP values are reported as presented in the original studies (e.g., study-level or subject-level totals, where applicable). For cross-study comparison, FP/day was used as the primary normalized metric when reported or derivable. FP/patient/day was not used uniformly because some studies, especially in-silico studies, reported only total evaluation days and not clearly defined patient-level monitoring time, making this conversion unreliable in some cases. The “Days” column represents the total evaluation duration used for normalization where applicable.*
