# Supplementary material for: On the road to fully automated insulin delivery: A systematic review of meal announcement free algorithms
Source: PLOS Digit Health. 2026 Jul 9;5(7):e0001492. doi: 10.1371/journal.pdig.0001492 (PMC13349122; doi:10.1371/journal.pdig.0001492)
Supplement: S2 Table — (DOCX) [file pdig.0001492.s003.docx]

**S2 Table. Glycemic outcomes with automated meal detection vs manual or no meal announcements.**

| **Ref – [Year]** | **Dataset** | **N** | **Data Used** | **Therapy** | **Category** | **MD** | **Method** | **Performance Metrics** |
| --- | --- | --- | --- | --- | --- | --- | --- | --- |
| Daniels et al. (9) – [2022] | In-silico  (UVA/Padova) | 10 | CGM, insulin | Insulin-only | Machine learning approach | Explicit add-on module | LSTM network-based seq2seq model | *CL with meal announcements:*  TBR: 1.5, TIR: 84.7, TAR: 13.7  *CL with meal detection:*  TBR: 1.4, TIR: 77.8, TAR: 20.7 |
| Mosquera- Lopez et al. (10) – [2023] | In-vivo | 15 | CGM, insulin, time | Insulin-only | Machine learning approach | Explicit add-on module | Multioutput neural network | Compared to hybrid CL MPC algorithm in a clinical trial, improvement was observed in TAR and TIR, without significant increase in TBR |
| Ibrahim et al. (11) – [2024] | In-silico  (UVA/Padova and Hovorka) | 20, 47 | CGM, insulin | Insulin-only | Machine learning approach | Explicit add-on module | Ensemble machine learning approach combining NN, LR and RF | When compared to OL-control without automated MD, significantly improvement in TIR and TAR was observed with minimal increase in hypoglycemia.  ***CL system with automated MD vs OL system***  ***1. Hovorka***  ***2. UVA*** |
| Xu et al. (12) – [2021] | In-silico  (UVA/Padova) | 30 | CGM | Insulin-only | Control system theory | Explicit add-on module | unscented KF (UKF) with decision rules | *Hybrid PID Control:* TBR: 1, TIR: 91.3, TAR: 7.6  *With automated MD:* TBR: 0, TIR: 96.6, TAR: 3.3 |
| Mahmoudi et al. (13) – [2018] | In-silico  (MVP model) | 9 | CGM, insulin | Insulin-only | Control system theory | Explicit add-on module | KF with CUSUM and threshold tests | *With manual meal announcement:*  TBR: 0, TIR: 85.6, TAR: 14.4  *With automated MD:*  TBR: 0, TIR: 71.6, TAR: 28.4 |
| Xie et al. (14) – [2017] | In-silico  (UVA/Padova) | 30 | CGM, insulin | Insulin-only | Control system theory | Explicit add-on module | Variable state dimension using KF | *Basal-bolus (BB) control (with meal announcements):*  TBR: 5, TIR: 85.5, TAR: 9.5  *BB control with automated MD (50% unannounced meals):*  TBR: 11, TIR: 73, TAR: 23 |
| Wang et al. (16) – [2010] | In-silico  (UVA/Padova) | 100 | CGM | Insulin-only | Heuristic approach | Explicit add-on module | Threshold based ROC of CGM (7) | *OL system (with meal announcements):*  TBR: 4, TIR: 82.7, TAR:13.3  *With Proposed automated MD:*  TBR: 0.9, TIR: 73.4, TAR:25.7 |
| Pimentel et al. (17) – [2020] | In-silico  (UVA/Padova) | 1 | CGM, insulin | Insulin-only | Heuristic approach | Explicit add-on module | Feedback scheme | *PID control (with automated MD)* TBR: 0, TIR: 90.7, TAR: 9.3  *PID control (manual mealtime boluses)* TBR: 0, TIR: 91.4, TAR: 8.6 |
| Lee et al. (19) – [2008] | In-silico  (Hovorka) | 1 | CGM | Insulin-only | Heuristic approach | Explicit add-on module | KF and rate-of-change of glucose concentration | *CL MPC (announced meals):* MAD: 91.0 g, Mean (Min, Max): 191.0 (96.6, 311.4) mg/dL  *CL MPC (unannounced meals):* MAD: 91.0 g, Mean (Min, Max): 191.0 (96.6, 311.4) mg/dL  *CL MPC (automated detected meals):* MAD: 38.6 g, Mean (Min, Max): 137.4 (87.3, 234.9) mg/dL |
| Mahmoudi et al. (20) – [2019] | In-silico  (Medtronic virtual patient - MVP) | 9 | CGM, insulin | Insulin-only | Control system theory | Explicit add-on module | Kalman filter (KF) along with CUSUM and threshold tests | *CL system (with announced meals):*  TBR-1: 0, TBR-2: 0, TIR: 91, TAR-1: 9, TAR-2: 0  *CL system (with MD):*  TBR-1: 0, TBR-2: 0, TIR: 83, TAR-1: 16, TAR-2: 0  When compared to glycemic control under no manual meal announcements |
| Harvey et al. (22) – [2014] | In-silico  (UVA/Padova) | 10 | CGM | Insulin-only | Heuristic approach | Explicit add-on module | GRID algorithm | When used automated MD along with zone-MPC  TBR: 0, TIR: 74, TAR-1: 25, TAR-2: 4.7 |
| Arpita et al. (23) – [2019] | In-silico  (UVA/Padova) | 10 | CGM | Insulin-only | Heuristic approach | Explicit add-on module | GRID algorithm proposed in (22) | *Conventional internal model control (IMC):*  TBR: 0.27, TIR: 62.49, TAR: 37.16  *Compound IMC (IMC + enhanced IMC):*  TBR: 0.17, TIR: 65.64, TAR: 34.17 |
| Sanz et al. (24) – [2017] | In-silico  (UVA/Padova) | 10 | CGM | Insulin-only | Heuristic approach | Explicit add-on module | GRID algorithm proposed in (22) | *Zone-MPC (with meal announcements):*  Mean CGM: 134 mg/dL, TIR: 91, TBR: 0  *Zone-MPC (with automated meal detection):*  Mean CGM: 149 mg/dL, TIR: 74.6, TBR: 0.16 |
| Atlas et al. (27) – [2010] | In-vivo | 7 | CGM, insulin | Insulin-only | Heuristic approach | Explicit add-on module | FL | *During 24 hour CL control*  TBR: 0, TIR: 73, TAR:27 |
| Dovc et al. (28) – [2020] | In-vivo | 20 | CGM, insulin | Insulin-only | Heuristic approach | Explicit add-on module | FL | During 27 hour observational period  *FAID + Faster insulin:*  TBR: 0, TIR: 53.8, TAR-2: 6.8  *FAID + Standard insulin:*  TBR: 0, TIR: 58.6, TAR-2: 8.7 |
| Samadi et al. (29) – [2017] | In-silico  (UVA/Padova) | 30 | CGM, insulin | Insulin-only | Heuristic approach | Explicit add-on module | Fuzzy logic (FL) | *Hybrid CL with announced meals:*  TBR: 4.1, TIR: 85.4, TAR: 10.4  *CL with automated meal detection:*  TBR: 3.1, TIR: 76.8, TAR: 20 |
| Richard et al. (31) – [2013] | In-vivo | 12 | CGM | Insulin-only | Heuristic approach | Implicit | FL | Only 7 patients completed the study:  Mean BG: 165.1 mg/dL,  TBR: 0.1, TIR: 65, TAR: 34.8 |
| Lim et al. (36) – [2023] | In-silico  (UVA/Padova) | 20 | CGM, CHO | Insulin-only | Machine learning approach | Explicit add-on module | Deep neural network | *CL MPC (with meal announcements):* TBR-1: 0.2, TBR-2: 0, TIR: 90.8, TAR-1: 9.1, TAR-2: 0  *CL MPC (with automated MD):* TBR-1: 0.7, TBR-2: 0, TIR: 85.2, TAR-1: 11.7, TAR-2: 1.4 |
| Lee et al. (45) – [2021] | In-silico  (UVA/Padova) | 20 | CGM, insulin, CHO | Insulin-only | Machine learning approach | Implicit | Reinforcement learning | Mean glucose: 124.72 mg/dL,  TBR: 1.07, TIR: 89.56, TAR: 9.52 |
| Sayyar et al. (46) – [2024] | In-silico  (UVA/Padova) | 67 | CGM, insulin | Insulin-only | Machine learning approach | Explicit add-on module | Deep reinforcement learning | *Proposed fully automated AID system:*  TBR: 0.9, TIR: 71.2, TAR: 30.8  *Hybrid AID system with CHO misestimation:*  TBR: 0.1, TIR: 76.2, TAR: 23.0  *Hybrid AID system with deep reinforcement:*  TBR: 0, TIR: 75.7, TAR: 21.4 |
| Sanz et al. (48) – [2020] | In-silico  (UVA/Padova) | 10 | CGM, insulin | Insulin-only | Control system theory | Implicit | Disturbance observer-based control with feed-forward strategy | *Hybrid CL with announced meals:*  TBR: 0, TIR: 93.3, TAR-2: 0.06  *CL with automated meal detection:*  TBR: 0, TIR: 85, TAR-2: 0.56 |
| Lee et al. (49) – [2014] | In-silico  (UVA/Padova) | 10 | CGM | Insulin-only | Control system theory | Implicit | Zone-MPC with Moving horizon estimator | *Zone-MPC with luenberger observer:* TIR: 54%  *Zone-MPC with moving horizon state estimator (MHSE):* TIR: 67% |
| Cai et al. (50) – [2022] | In-silico  (UVA/Padova) | 10 | CGM | Insulin, glucagon | Control system theory | Implicit | Extended state observer and adaptive feedback mechanisms | Proposed controller performance  *With announced meals:*  TBR: 0, TIR: 94.1, TAR-1: 5.9, TAR-2: 0  *With unannounced meals*  TBR: 0, TIR: 75.1, TAR-1: 24.9, TAR-2: 1.5 |
| Sala-Mira et al. (51) – [2019] | In-silico  (UVA/Padova) | 30 | CGM | Insulin-only | Control system theory | Explicit add-on module | Super-twisting based residual generator | *Hybrid AID (with announced meals)*:  TBR: 0, TIR: 86.9, TAR-1: 13, TAR-2: 0  *Meal-announcement free AID*:  TBR: 0, TIR: 78.9, TAR-1: 20.9, TAR-2: 2.3 |
| Sala-Mira et al. (52) – [2022] | In-silico  (UVA/Padova) | 10 | CGM | Insulin-only | Control system theory | Explicit add-on module | Super-twisting based residual generator | *PID Hybrid CL (with announcements):*  TBR: 0, TIR: 84, TAR: 17.2  *PID Hybrid CL + automated MD:*  TBR: 0, TIR: 81.9, TAR: 19.2 |
| Corbett et al. (54) – [2022] | In-silico  (UVA/Padova) | 100 | CGM, insulin | Insulin-only | Control system theory | Explicit add-on module | Anticipation and automatic blousing using MPC and KF | Proposed *Multistage-MPC + bolus priming*  TBR: 1.12, TIR: 77.17, TAR-1: 22.18, TAR-2: 10.45 |
| Garcia-Tirado et al. (55) – [2021] | In-silico  (UVA/Padova) | 100 | CGM, insulin | Insulin-only | Control system theory | Explicit add-on module | Bolus priming system with KF & Model Predictive Control (MPC) | *Proposed RocketAP (80 g unannounced meal):*  TBR: 0, TIR: 76.1, TAR-1: 23.8, TAR-2: 0 |
| Garcia et al. (56) – [2021] | In-vivo | 18 | CGM | Insulin-only | Control system theory | Implicit | MPC  (RocketAP) | *Proposed fully CL (with automated MD):*  TBR: 0, TIR: 83, TAR: 17 |
| Song et al. (57) – [2020] | In-vivo | 29 | CGM, insulin | Insulin-only | Control system theory | Implicit | MPC | *CL with automated meal detection:* TBR: 0, TIR: 65.2, TAR:32.1 |
| Cameron et al. (58) – [2009] | In-silico  (UVA/Padova) | 100 | CGM, insulin | Insulin-only | Control system theory | Explicit add-on module | MMPPC and evolving framework (analysis on the ROC of residuals) | *CL without MD:* Mean BG: 137 mg/dL, TBR: 0, TIR: 87, TAR: 13.4  *CL with MD:* Mean BG: 132 mg/dL, TBR: 0, TIR: 89, TAR: 11.4 |
| Cameron et al. (59) – [2014] | In-vivo | 10 | CGM | Insulin-only | Control system theory | Implicit | MMPPC | Average glycemic control results:  TBR: 1.31, TIR: 71.1, TAR-2: 2.51 |
| Cameron et al. (60) – [2017] | In-vivo | 25 | CGM, insulin, accelerometer | Insulin-only | Control system theory | Implicit | Multiple model probabilistic predictive control (MMPPC) | *Overall CL 24h:*  Mean: 147 mg/dL, TBR-1: 1.75, TBR-2: 0.08, TIR: 75.5, TAR-1: 17, TAB-2: 7.35 |
| Harvey et al. (61) – [2014] | In-vivo | 12 | CGM | Insulin-only | Control system theory | Implicit | Zone-MPC | Average glycemic control results:  TBR: 2.2, TIR: 71.2, TAR: 28.8 |
| Cameron et al. (62) – [2011] | In-silico  (UVA/Padova) | 10 | CGM, insulin | Insulin-only | Control system theory | Explicit add-on module | Extended MPC with a risk management framework | Glycemic control performance  *Proposed enhanced MPC:*  TBR: 0.7, TIR: 84.3, TAR-1: 15, TAR-2: 0 |
| Turksoy et al. (65) – [2017] | In-vivo | 7 | CGM | Insulin-only | Control system theory | Explicit add-on module | UKF | *CL with MD:*  TBR: 2.2, TIR: 70.7, TAR: 27.3 |
| Carlos et al. (69) – [2022] | In-silico  (UVA/Padova) | 10 | CGM, insulin, CHO | Insulin-only | Control system theory | Explicit add-on module | KF with Feedback scheme-based algorithm | *CL MPC + automated MD:*  TBR-1: 0, TBR-2: 0, TIR: 94.1, TAR-1: 5.6, TAR-2: 0 |
| Fathi et al. (74) – [2019] | In-vivo | 4 | CGM, insulin | Insulin-only | Control system theory | Explicit add-on module | KF | Area Under the Curves (AUCs) comparison:  (unit is *“h mmol/L”*)  *Conventional insulin therapy*: AUC: 29.6 ± 6.5  *CL therapy*: AUC: 24.8 ± 11.5  *CL therapy with meal detection*: AUC: 18.0 ± 2.7 |
| Majdpour et al. (75) – [2021] | In-vivo | 9 | CGM, insulin | Insulin, glucagon, pramlintide | Control system theory | Explicit add-on module | KF with decision rule | *Insulin-only CL + MD:*  TBR: 4.7, TIR: 83.3, TAR: 12  *Multihormone CL + MD:*  TBR: 0.93, TIR: 81, TAR: 18 |
| Fushimi et al. (77) – [2019] | In-silico  (UVA/Padova) | 10 | CGM | Insulin-only | Control system theory | Explicit add-on module | SSG with ARG algorithm proposed in (76) | *CL system (with announced meals):*  TBR: 0, TIR: 83.3, TAR: 16.6  *CL system (with automated MD):*  TBR: 0, TIR: 85, TAR: 14.8 |
| Fushimi et al. (78) – [2020] | In-silico  (UVA/Padova) | 20 | CGM | Insulin-only | Control system theory | Explicit add-on module | SSG with modified ARG proposed in (76) | *CL system (with announced meals):*  TBR: 0, TIR: 79, TAR: 20.7  *CL system (with automated MD):*  TBR: 0, TIR: 77.7, TAR: 22.2 |
| Palisaitis et al. (79) – [2021] | In-vivo | 11 | CGM | Insulin-only | Control system theory | Explicit add-on module | Predictive models with threshold tests | 4-h postprandial performance for unannounced meal  *AID + MD:* TBR: 0, TIR: 40.9, TAR: 58 |
| Turksoy et al. (80) – [2013] | In-vivo | 3 | CGM, plasma, insulin-ion-board, galvanic skin response | Insulin-only | Control system theory | Implicit | Multivariable adaptive control | Average glycemic control results:  TBR: 1.6, TIR: 62.1, TAR-1: 27.1, TAR-2: 9.1 |
| Abbes et al. (81) – [2013] | In-silico  (UVA/Padova) | 10 | CGM | Insulin-only | Control system theory | Implicit | Proportional Integral derivative (PID) controller | With unannounced meals  Mean BG: 135 mg/dL, TBR-2: 0.8, TBR-1: 6, TIR: 77, TAR-1: 16, TAR-2: 0.3 |
| Rodriguez et al. (82) – [2024] | In-silico  (UVA/Padova) | 10 | CGM, insulin | Insulin-only | Control system theory | Implicit | Markovian controller (using KF) | *Glycemic control performance:*  TBR: 0.1, TIR: 71.8, TAR: 28.1 |

*N – number of patients; MD – meal detection; Min – minimum; Max – maximum; OL – open-loop system; CL – closed-loop system; FAID – fully closed-loop automated insulin delivery system; CUSUM – cumulative sum;* *LSTM – long short-term memory; TIR – time-in-range (70-180 mg/dL); TBR-1 – time-below-range level 1 (70-54 mg/dL); TBR-2 – time-below-range level 2 (<54 mg/dL); TAR-1 – time-above-range level 1 (180-250 mg/dL); TAR-2 – time-above-range level 2 (>250 mg/dL);* *CGM – continuous glucose monitoring; CHO – carbohydrates intake information; HR – heart rate; SVM – support vector machine; NB – naïve bayes; NN – neural network; LR – logistic regression; RF – random forest; PA – physical activity information; LDA – linear discriminant analysis; MAD – mean absolute deviation.*

- *Citation numbers are consistent with those in the main manuscript.*
