# Supplementary material for: On the road to fully automated insulin delivery: A systematic review of meal announcement free algorithms
Source: PLOS Digit Health. 2026 Jul 9;5(7):e0001492. doi: 10.1371/journal.pdig.0001492 (PMC13349122; doi:10.1371/journal.pdig.0001492)
Supplement: S3 Table — (DOCX) [file pdig.0001492.s004.docx]

**S3 Table. Data partitioning, validation, and performance reporting in machine learning-based meal detection studies**

| **Ref - [Year]** | **Dataset** | **N** | **Partitioning Scheme** | **Training / Testing Split** | **Validation setting** | **Cross-validation (CV)** | **Performance Metrics** |
| --- | --- | --- | --- | --- | --- | --- | --- |
| Daniels et al. (9) - [2022] | in-silico | 10 | Aggregated pretraining +  subject-specific fine-tuning | 80% / 20% (within each subject);  Total days: 60 days | Retrospective | No | Sens: 76%, Pre: 93%, F1-Score: 84%, DT: 38 min |
| Mosquera et al. (10) - [2023] | in-vivo | 13 | In-silico pretraining;  fixed model evaluated clinically | Not applicable | Prospective | Yes (CV for hyperparameter tuning; method not specified) | In-silico validation: Sens: 79 to 90%, DT: 27.5 ± 4.8 min With real T1D subjects: Sens: 83.3%, FP/Day: 1.0, DT: 25.9 ± 0.9 min |
| Ibrahim et al. (11) - [2024] | in-silico + in-vivo | 20+47; 12 | Subject-wise partitioning (in-silico); personalized models | In-silico: separate training (120 days) and testing (30 days) datasets per subject; OhioT1DM: leave-one-patient-out (11 train / 1 test per fold) | Retrospective | Yes (5-fold stratified CV for  hyperparameter tuning; leave-on-patient-out for OhioT1DM) | In-silico:  Sens : 74.5-95%, Pre : 83.5-96%, F1-score : 82-88.5, FP/day: 0.05-0.31, DT: 21-31 min  In-vivo (OhioT1DM) :  Sens: 47-71%, Pre: 62-83%, F1-Score: 60-66%, FP/Day: 0.07-0.32, DT: 33-43 min |
| Maria et al. (34) - [2021] | in-silico | 30 | Subject-wise partitioning | 70% / 30% split of simulated days per subject (same subjects in train and test);  Total days: 7 days per subject | Retrospective | Yes (validation split (15% of training dataset used for model selection) | Sens: 73.80-74.62%, Spe: 69.27-71.49%, Acc: 70.48-72.24%, DT: 8.95-9.71 min |
| Lu et al. (35) - [2024] | in-vivo | 82 | Temporal split within subject (per-day) | 70% / 10% / 20% time-based split within subject (training / validation / testing; same subjects in all sets);  Total days: 10497 days | Retrospective | No | Sens: 97.72% ; Pre: 93.51%; F1-Score: 95.33%; FP/Day: 0.25; FN/Day: 0.07 |
| Lim et al. (36) - [2023] | in-silico | 20 | Online adaptive learning (continual within-subject updating) | No fixed train/test split; first day used for initialization, subsequent days used for online updating and evaluation;  Total days: 10 days | Retrospective | No | Not reported |
| Askari et al. (37) - [2022] | in-vivo | 11 | Subject-wise personalized modeling | Within-subject split of samples; 87.5% used for training and 12.5% used for testing;  Total days: 4315 days | Retrospective | Yes (stratified 6-fold CV on training data) | LSTM NN: Acc: 88.64-92.32%, Sens: 88.64-92.32%, Pre: 95.25-96.17%, F1-Score: 91.40-94.00% |
| Fazakas et al. (38) - [2024] | in-vivo | 351 | Temporal within-subject partitioning (per day) | 80% / 20% time-ordered split within each day (earlier data for training, later data for testing);  Total days: 354 days | Retrospective | No | Sens: 98.8%; Pre: 99%; F1-Score: 98.9%,  DT: 5 to 6 seconds |
| Zheng et al. (39) - [2020] | in-silico | 4 | Subject-wise partitioning | Fixed training  (5 days) + testing (20 days) | Retrospective | No | Sens: 90%, FP rate: 11.47%, FP/Day: 0.39, DT: 35.1 min |
| Federico et al. (42) - [2023] | in-silico | 30 | Subject-wise partitioning | Leave-one-subject-out;  29 training/validation, 1 testing per fold | Retrospective | Yes (leave-one-subject-out with inner stratified CV) | Sens: 73.6%; Pre: 70%; F1-Score: 69.3% |
| Kolle et al. (43) - [2019] | in-vivo | 11 | Meal-wise leave-one-out validation | Leave-one-meal-out: classifiers trained on 9 meals and tested on 1 meal per iteration (same subjects may appear in train and test across folds) | Retrospective | Yes (leave-one-out CV on meals) | Sens: 40-50%, FP/Day: 0.08, DT: 10 min |
| Carvalho et al. (44) - [2023] | in-vivo | 12 | Subject-wise personalized modeling | Predefined temporal split per subject following OhioT1DM protocol; approximately 50% training, 25% validation, 25% testing (time-ordered within subject) | Retrospective | Yes (model selection across multiple classifiers using a held-out validation set per subject) | Sens: 59; Pre: 45; F1-Score: 50, FP: 19.2 |
| Lee et al. (45) - [2021] | in-silico | 20 | Subject-wise personalized reinforcement learning | No fixed train/test split; policies trained per subject using repeated one-day single-meal simulations and evaluated on separate one-day and multi-day scenarios | Retrospective | No | Not reported |
| Sayyar et al. (46) - [2024] | in-silico | 68 | Subject-wise personalized deep reinforcement learning | No fixed train/test split; agents trained per subject (1500 days) using long-horizon simulation (experience replay), evaluated on separate 14-day simulation scenarios | Retrospective | No | Sens: 64.3%, Pre: 89.9%, F1-Score: 74.93%, FP/Day: 0.29, and DT: 34.1 min |

*This table summarizes data partitioning schemes, training and testing strategies, validation settings, cross-validation approaches, and reported detection performance for machine learning–based meal detection studies.* ***Subject-wise partitioning*** *indicates that data are organized and modeled at the subject level, typically using personalized models or subject-specific splits; depending on the study, the same subject may contribute data to both training and testing through temporal or sample-level separation.* ***Temporal (time-based) splits*** *indicate that data are divided by time within the same subject, with earlier data used for training and later data used for testing. Reported durations (days) refer to the total simulated or recorded time used for model development and evaluation.* ***Validation setting*** *denotes whether evaluation was conducted retrospectively (offline analysis of simulated or previously collected data) or prospectively (real-time or clinical evaluation).* ***Cross-validation*** *entries distinguish between use for performance evaluation and use for hyperparameter tuning when explicitly reported. Studies based on reinforcement or online learning do not employ fixed train/test splits; performance is evaluated on separate simulation scenarios or later time periods. False-positive values are reported as presented in the original studies; when reported or derivable, FP/day is shown as the primary normalized metric for cross-study comparability, whereas FP/patient/day was not applied uniformly because some studies did not report clearly defined cumulative patient-level monitoring time.*
