# Supplementary material for: On the road to fully automated insulin delivery: A systematic review of meal announcement free algorithms
Source: PLOS Digit Health. 2026 Jul 9;5(7):e0001492. doi: 10.1371/journal.pdig.0001492 (PMC13349122; doi:10.1371/journal.pdig.0001492)
Supplement: S4 Table — (DOCX) [file pdig.0001492.s005.docx]

**S4 Table. Overview of automated meal detection studies and reported outcomes**

| **Ref** | **Year** | **Objective or innovation** | **Dataset type** | | **Methodology** | | | **Reported outcomes** | | | **Reported outcomes** | | |
| --- | --- | --- | --- | --- | --- | --- | --- | --- | --- | --- | --- | --- | --- |
|  |  |  | **In-silico** | **In-vivo** | **A** | **B** | **C** | **SE** | **FP/day** | **DT** | **TIR** | **TAR** | **TBR** |
| (7) | 2008 | CGM-based meal detection for AID system | **✘** | **✔︎** | **✔︎** | **✘** | **✘** | **✘** | **✘** | **✔︎** | **✘** | **✘** | **✘** |
| (8) | 2018 | UKF-based unannounced meal detection with tunings | **✔︎** | **✘** | **✘** | **✘** | **✔︎** | **✔︎** | **✔︎** | **✔︎** | **✘** | **✘** | **✘** |
| (9) | 2022 | Deep learning meal detection and CHO estimation | **✔︎** | **✘** | **✘** | **✔︎** | **✘** | **✔︎** | **✘** | **✔︎** | **✔︎** | **✔︎** | **✔︎** |
| (10) | 2023 | ML-based meal detection in robust AID | **✔︎** | **✔︎** | **✘** | **✔︎** | **✘** | **✔︎** | **✔︎** | **✔︎** | **✔︎** | **✔︎** | **✔︎** |
| (11) | 2024 | Ensemble ML meal detector with insulin compensation | **✔︎** | **✔︎** | **✘** | **✔︎** | **✘** | **✔︎** | **✔︎** | **✔︎** | **✔︎** | **✔︎** | **✔︎** |
| (12) | 2021 | UKF-based meal estimation with postprandial control | **✔︎** | **✘** | **✘** | **✘** | **✔︎** | **✔︎** | **✔︎** | **✔︎** | **✔︎** | **✔︎** | **✔︎** |
| (13) | 2018 | Kalman-filter–based meal detection with bolus calculator | **✔︎** | **✘** | **✘** | **✘** | **✔︎** | **✔︎** | **✔︎** | **✔︎** | **✔︎** | **✔︎** | **✔︎** |
| (14) | 2017 | Variable state dimension meal detection and estimation | **✔︎** | **✘** | **✘** | **✘** | **✔︎** | **✔︎** | **✔︎** | **✔︎** | **✔︎** | **✔︎** | **✔︎** |
| (16) | 2010 | Automatic bolus and adaptive basal algorithm | **✔︎** | **✘** | **✔︎** | **✘** | **✘** | **✘** | **✘** | **✘** | **✔︎** | **✔︎** | **✔︎** |
| (17) | 2020 | Modular intelligent AID controller architecture | **✔︎** | **✘** | **✔︎** | **✘** | **✘** | **✘** | **✘** | **✔︎** | **✔︎** | **✔︎** | **✔︎** |
| (18) | 2009 | MPC with sliding meal size estimator | **✔︎** | **✘** | **✔︎** | **✘** | **✘** | **✔︎** | **✔︎** | **✔︎** | **✘** | **✘** | **✘** |
| (19) | 2008 | MPC with automatic meal disturbance rejection | **✔︎** | **✘** | **✔︎** | **✘** | **✘** | **✘** | **✘** | **✔︎** | **✔︎** | **✔︎** | **✔︎** |
| (20) | 2019 | KF-based meal detection + CHO estimation + bolus | **✔︎** | **✘** | **✘** | **✘** | **✔︎** | **✔︎** | **✘** | **✔︎** | **✔︎** | **✔︎** | **✔︎** |
| (21) | 2020 | Pattern-recognition–based meal detection without individual tuning | **✘** | **✔︎** | **✔︎** | **✘** | **✘** | **✔︎** | **✔︎** | **✔︎** | **✘** | **✘** | **✘** |
| (22) | 2014 | Glucose Rate Increase Detector (GRID) for safe meal-triggered bolus | **✔︎** | **✔︎** | **✔︎** | **✘** | **✘** | **✔︎** | **✔︎** | **✔︎** | **✔︎** | **✔︎** | **✔︎** |
| (23) | 2019 | Online-tuned model-based compound controller with meal detection | **✔︎** | **✘** | **✔︎** | **✘** | **✘** | **✘** | **✘** | **✘** | **✔︎** | **✔︎** | **✔︎** |
| (24) | 2017 | Adaptive MPC with confidence index and meal-triggered bolus | **✔︎** | **✘** | **✔︎** | **✘** | **✘** | **✘** | **✘** | **✘** | **✔︎** | **✔︎** | **✔︎** |
| (25) | 2019 | Simulation-driven meal detection using CGM and activity data | **✔︎** | **✔︎** | **✔︎** | **✘** | **✘** | **✔︎** | **✔︎** | **✔︎** | **✘** | **✘** | **✘** |
| (26) | 2016 | Physiology-invariant meal detection (PAIN) | **✘** | **✔︎** | **✔︎** | **✘** | **✘** | **✔︎** | **✔︎** | **✘** | **✘** | **✘** | **✘** |
| (27) | 2010 | MD-Logic fuzzy-logic artificial pancreas | **✘** | **✔︎** | **✔︎** | **✘** | **✘** | **✘** | **✘** | **✔︎** | **✔︎** | **✔︎** | **✔︎** |
| (28) | 2020 | FAID control under unannounced meals | **✘** | **✔︎** | **✔︎** | **✘** | **✘** | **✘** | **✘** | **✔︎** | **✔︎** | **✔︎** | **✔︎** |
| (29) | 2017 | Fuzzy-logic meal detection and CHO estimation | **✔︎** | **✘** | **✔︎** | **✘** | **✘** | **✔︎** | **✔︎** | **✔︎** | **✔︎** | **✔︎** | **✔︎** |
| (30) | 2018 | Automated detection and estimation of unannounced meals in multivariable AP | **✘** | **✔︎** | **✔︎** | **✘** | **✘** | **✔︎** | **✔︎** | **✔︎** | **✘** | **✘** | **✘** |
| (31) | 2013 | FAID fuzzy-logic controller without meal announcement | **✘** | **✔︎** | **✔︎** | **✘** | **✘** | **✘** | **✘** | **✘** | **✔︎** | **✔︎** | **✔︎** |
| (32) | 2019 | FAID with meal detection and bolusing | **✘** | **✔︎** | **✔︎** | **✘** | **✘** | **✔︎** | **✔︎** | **✔︎** | **✘** | **✘** | **✘** |
| (34) | 2021 | LSTM-based automated meal detection from CGM | **✔︎** | **✘** | **✘** | **✔︎** | **✘** | **✔︎** | **✘** | **✔︎** | **✘** | **✘** | **✘** |
| (35) | 2024 | Personalized mealtime prediction using insulin pump data | **✘** | **✔︎** | **✘** | **✔︎** | **✘** | **✔︎** | **✔︎** | **✘** | **✘** | **✘** | **✘** |
| (36) | 2023 | Learning-based MPC with meal detection and estimation | **✔︎** | **✘** | **✘** | **✔︎** | **✘** | **✘** | **✘** | **✘** | **✔︎** | **✔︎** | **✔︎** |
| (37) | 2022 | Meal and physical activity detection from free-living data | **✘** | **✔︎** | **✘** | **✔︎** | **✘** | **✔︎** | **✘** | **✘** | **✘** | **✘** | **✘** |
| (38) | 2024 | Food intake detection using Inertial Measurement Unit (IMU) sensors and deep learning | **✘** | **✔︎** | **✘** | **✔︎** | **✘** | **✔︎** | **✘** | **✔︎** | **✘** | **✘** | **✘** |
| (39) | 2020 | Unannounced meal detection using Extended Isolation Forest | **✔︎** | **✘** | **✘** | **✔︎** | **✘** | **✔︎** | **✔︎** | **✔︎** | **✘** | **✘** | **✘** |
| (42) | 2023 | AI-based comparison of meal detection strategies across age groups | **✔︎** | **✘** | **✘** | **✔︎** | **✘** | **✔︎** | **✘** | **✘** | **✘** | **✘** | **✘** |
| (43) | 2019 | Early meal detection using abdominal sound signals | **✘** | **✔︎** | **✘** | **✔︎** | **✘** | **✔︎** | **✔︎** | **✔︎** | **✘** | **✘** | **✘** |
| (44) | 2023 | Data-driven personalized meal detection from CGM patterns | **✘** | **✔︎** | **✘** | **✔︎** | **✘** | **✔︎** | **✘** | **✘** | **✘** | **✘** | **✘** |
| (45) | 2021 | FAID using bio-inspired reinforcement learning | **✔︎** | **✘** | **✘** | **✔︎** | **✘** | **✘** | **✘** | **✘** | **✔︎** | **✔︎** | **✔︎** |
| (46) | 2024 | Deep RL bolus calculator for unannounced meals | **✔︎** | **✘** | **✘** | **✔︎** | **✘** | **✔︎** | **✔︎** | **✔︎** | **✔︎** | **✔︎** | **✔︎** |
| (48) | 2020 | Disturbance-observer-based AID with feed-forward compensation | **✔︎** | **✘** | **✘** | **✘** | **✔︎** | **✘** | **✘** | **✘** | **✔︎** | **✔︎** | **✔︎** |
| (49) | 2014 | Zone-MPC with moving-horizon estimation for meal disturbance | **✔︎** | **✘** | **✘** | **✘** | **✔︎** | **✘** | **✘** | **✘** | **✔︎** | **✘** | **✘** |
| (50) | 2022 | Event-triggered active disturbance rejection dual-hormone AID | **✔︎** | **✘** | **✘** | **✘** | **✔︎** | **✘** | **✘** | **✘** | **✔︎** | **✔︎** | **✔︎** |
| (51) | 2019 | Sliding-mode disturbance observers for unannounced meals | **✔︎** | **✘** | **✘** | **✘** | **✔︎** | **✔︎** | **✔︎** | **✔︎** | **✔︎** | **✔︎** | **✔︎** |
| (52) | 2022 | IMC-based module removing meal and exercise announcements | **✔︎** | **✘** | **✘** | **✘** | **✔︎** | **✘** | **✘** | **✘** | **✔︎** | **✔︎** | **✔︎** |
| (53) | 2022 | Super-twisting-based meal detector evaluated on real-life data | **✘** | **✔︎** | **✘** | **✘** | **✔︎** | **✔︎** | **✔︎** | **✔︎** | **✘** | **✘** | **✘** |
| (54) | 2022 | Online disturbance rejection and anticipatory bolusing for FAID | **✔︎** | **✘** | **✘** | **✘** | **✔︎** | **✘** | **✘** | **✘** | **✔︎** | **✔︎** | **✔︎** |
| (55) | 2021 | Advanced hybrid MPC with automatic bolus priming for unannounced meals | **✔︎** | **✘** | **✘** | **✘** | **✔︎** | **✘** | **✘** | **✔︎** | **✔︎** | **✔︎** | **✔︎** |
| (56) | 2021 | Learning-based MPC for unannounced meals | **✘** | **✔︎** | **✘** | **✘** | **✔︎** | **✘** | **✘** | **✘** | **✔︎** | **✔︎** | **✔︎** |
| (57) | 2020 | MPC based FAID with meal detection | **✘** | **✔︎** | **✘** | **✘** | **✔︎** | **✘** | **✘** | **✘** | **✔︎** | **✔︎** | **✔︎** |
| (58) | 2009 | Inpatient trial of multiple model probabilistic predictive control (MMPPC) with repeated unannounced meals | **✔︎** | **✘** | **✘** | **✘** | **✔︎** | **✘** | **✘** | **✘** | **✔︎** | **✔︎** | **✔︎** |
| (59) | 2014 | FAID without meal announcement (MMPPC) | **✘** | **✔︎** | **✘** | **✘** | **✔︎** | **✘** | **✘** | **✘** | **✔︎** | **✔︎** | **✔︎** |
| (60) | 2017 | Zone-MPC with health monitoring under unannounced meals | **✘** | **✔︎** | **✘** | **✘** | **✔︎** | **✘** | **✘** | **✘** | **✔︎** | **✔︎** | **✔︎** |
| (61) | 2014 | Risk-based MPC for FAID | **✘** | **✔︎** | **✘** | **✘** | **✔︎** | **✘** | **✘** | **✘** | **✔︎** | **✔︎** | **✔︎** |
| (62) | 2011 | UKF-based meal detection module for multivariable AID | **✔︎** | **✘** | **✘** | **✘** | **✔︎** | **✘** | **✘** | **✘** | **✔︎** | **✔︎** | **✔︎** |
| (64) | 2016 | Meal detection and bolusing in adaptive AID using UKF | **✘** | **✔︎** | **✘** | **✘** | **✔︎** | **✔︎** | **✔︎** | **✘** | **✘** | **✘** | **✘** |
| (65) | 2017 | Real-time meal detection and bolusing using UKF | **✘** | **✔︎** | **✘** | **✘** | **✔︎** | **✘** | **✘** | **✘** | **✔︎** | **✔︎** | **✔︎** |
| (66) | 2017 | Meal detection robustness during exercise | **✔︎** | **✘** | **✘** | **✘** | **✔︎** | **✔︎** | **✘** | **✔︎** | **✘** | **✘** | **✘** |
| (68) | 2021 | Feedback-based meal detection and CHO estimation | **✔︎** | **✔︎** | **✘** | **✘** | **✔︎** | **✔︎** | **✔︎** | **✔︎** | **✘** | **✘** | **✘** |
| (69) | 2022 | Meal detection coupled with zone MPC | **✔︎** | **✘** | **✘** | **✘** | **✔︎** | **✔︎** | **✔︎** | **✔︎** | **✔︎** | **✔︎** | **✔︎** |
| (70) | 2018 | MHE-based unannounced meal detection | **✔︎** | **✘** | **✘** | **✘** | **✔︎** | **✔︎** | **✔︎** | **✔︎** | **✘** | **✘** | **✘** |
| (71) | 2019 | Committed MHE for meal detection and estimation | **✔︎** | **✘** | **✘** | **✘** | **✔︎** | **✔︎** | **✔︎** | **✔︎** | **✘** | **✘** | **✘** |
| (72) | 2017 | Fault and meal detection using redundant CGMs and UKF | **✔︎** | **✘** | **✘** | **✘** | **✔︎** | **✔︎** | **✘** | **✔︎** | **✘** | **✘** | **✘** |
| (73) | 2020 | Model-based detection of missed meal announcements | **✔︎** | **✘** | **✘** | **✘** | **✔︎** | **✔︎** | **✔︎** | **✔︎** | **✘** | **✘** | **✘** |
| (74) | 2019 | Model-based unannounced meal detection using KF | **✔︎** | **✔︎** | **✘** | **✘** | **✔︎** | **✔︎** | **✔︎** | **✔︎** | **✘** | **✘** | **✘** |
| (75) | 2021 | Multihormone FAID with model-based meal detection | **✘** | **✔︎** | **✘** | **✘** | **✔︎** | **✘** | **✘** | **✔︎** | **✔︎** | **✔︎** | **✔︎** |
| (76) | 2019 | ARG algorithm with automatic switching signal generator | **✔︎** | **✔︎** | **✘** | **✘** | **✔︎** | **✔︎** | **✔︎** | **✔︎** | **✘** | **✘** | **✘** |
| (77) | 2019 | ARG without meal announcement | **✔︎** | **✘** | **✘** | **✘** | **✔︎** | **✘** | **✘** | **✘** | **✔︎** | **✔︎** | **✔︎** |
| (78) | 2020 | Switched control AID for pediatric population | **✔︎** | **✘** | **✘** | **✘** | **✔︎** | **✘** | **✘** | **✘** | **✔︎** | **✔︎** | **✔︎** |
| (79) | 2021 | Clinical trial of meal detection algorithm in adolescents | **✘** | **✔︎** | **✘** | **✘** | **✔︎** | **✘** | **✘** | **✘** | **✔︎** | **✔︎** | **✔︎** |
| (80) | 2013 | Multivariable adaptive control without meal announcement | **✘** | **✔︎** | **✘** | **✘** | **✔︎** | **✘** | **✘** | **✘** | **✔︎** | **✔︎** | **✔︎** |
| (81) | 2013 | PID-based AID under unannounced meals | **✔︎** | **✘** | **✘** | **✘** | **✔︎** | **✘** | **✘** | **✘** | **✔︎** | **✔︎** | **✔︎** |
| (82) | 2024 | Robust Markovian control for FAID | **✔︎** | **✘** | **✘** | **✘** | **✔︎** | **✘** | **✘** | **✘** | **✔︎** | **✔︎** | **✔︎** |

*In-silico indicates simulation-based evaluation using virtual patient models; In-vivo indicates evaluation using clinical or real-world data. A denotes heuristic-based methods (e.g., threshold rules, derivatives, GRID, fuzzy logic, or physiology-based rules); B denotes machine-learning–based methods (e.g., neural networks, ensemble learning, deep learning, or reinforcement learning); C denotes control-theory–based methods (e.g., Kalman filtering, moving-horizon estimation, model predictive control, disturbance observers, or proportional–integral–derivative control). SE: sensitivity; FP/day: false positives per day; DT: detection time; TIR: time in range (70–180 mg/dL); TAR: time above range (>180 mg/dL); TBR: time below range (<70 mg/dL). ✔︎ indicates that the metric or category was explicitly reported or applied in the corresponding study; ✘ indicates that it was not reported or not applicable.*

*AID - automated insulin delivery; FAID - fully automated insulin delivery; MPC - model predictive control; PID - proportional-integral-derivative control; ML - machine learning; LSTM - long short-term memory; CGM - continuous glucose monitoring; KF – Kalman filter; UKF – unscented Kalman filter; ARG - automated regulation of glucose; IMC – internal model control; MHE – moving horizon estimation*
