# Supplementary material for: On the road to fully automated insulin delivery: A systematic review of meal announcement free algorithms
Source: PLOS Digit Health. 2026 Jul 9;5(7):e0001492. doi: 10.1371/journal.pdig.0001492 (PMC13349122; doi:10.1371/journal.pdig.0001492)
Supplement: S1 Fig — (DOCX) [file pdig.0001492.s006.docx]

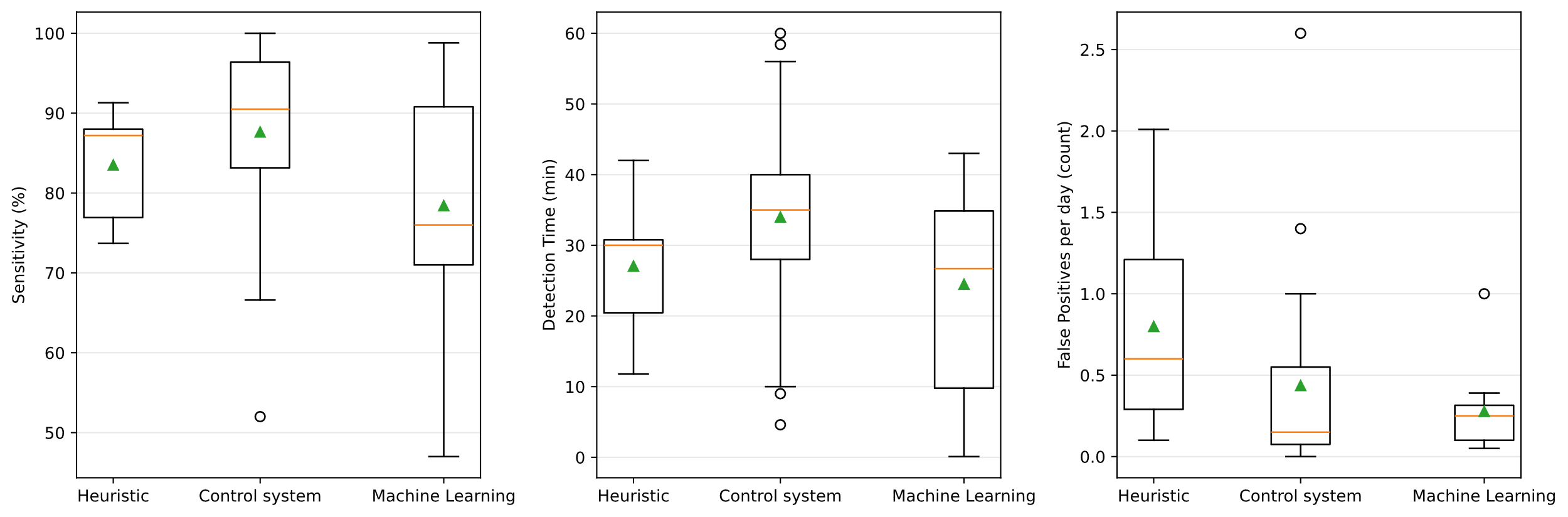


**S1 Fig. Distribution of reported performance metrics across automated meal detection method categories. In each box plot, the orange horizontal line indicates the median, the green triangle indicates the mean, and the black circles represent outliers. Boxes show the interquartile range, and whiskers indicate the spread of non-outlier values.**
